# Optimality Conditions for Cell-Fate Heterogeneity That Maximize the Effects of Growth Factors in PC12 Cells

**DOI:** 10.1371/journal.pcbi.1003320

**Published:** 2013-11-14

**Authors:** Kazunari Mouri, Yasushi Sako

**Affiliations:** Cellular Informatics Laboratory, RIKEN, 2-1 Hirosawa, Wako 351-0198, Saitama, Japan; Princeton University, United States of America

## Abstract

Recently, the heterogeneity that arises from stochastic fate decisions has been reported for several types of cancer-derived cell lines and several types of clonal cells grown under constant environmental conditions. However, the relation between this stochasticity and the responsiveness to extracellular stimuli remains largely unknown. Here we focused on the fate decisions of the PC12 cell line, which was derived from rat pheochromocytoma, and is a model system to study differentiation into sympathetic neurons. Whereas epidermal growth factor (EGF) stimulates the proliferation of populations of PC12 cells, nerve growth factor (NGF) promotes the differentiation of neurites to neuron-like cells. We found that phenotypic heterogeneity increased with time at several surrounding serum concentrations, suggesting stochastic cell-fate decisions in single cells. We made a simple mathematical model assuming Markovian transitions of the cell fates, and estimated the transition rates based on Bayes' theorem. The model suggests that depending on the serum concentration, EGF (NGF) even directs differentiation (proliferation) at the single-cell level. The maximum effects of the growth factors were ensured when the transition rates were appropriately controlled by the serum concentration to produce a nonextremal, moderate amount of cell-fate heterogeneity. Our model was validated by the experimental finding that the means and variances of the local cell densities obey a power-law relationship. These results suggest that even when efficient responses to growth factors are observed at the population level, the growth factors stochastically direct the cell-fate decisions in different directions at the single-cell level.

## Author Summary

Elucidation of the mechanisms that regulate cell fate has become one of the primary goals of research in cell biology and regenerative medicine. Growth factors are often used to regulate cell fate. However, stochastic cellular responses to growth regulators have prevented precise control of cell fate. We report our investigation of the relationship between heterogeneity and responsiveness in cell fate decisions by both single cells and populations of cells. Our study involved PC12, a cultured cell line for which cell-fates are affected by exposure to growth factors and culture conditions. Computational methods using a mathematical model enabled us to determine the cell-fate decisions rate in single PC12 cells and analyze the population responses to growth factors from experimental data. Our findings reveal that growth factors control cell-fate decisions rate in single PC12 cells, and suggest distinct differences in the mechanisms of actions of growth factors under different culture conditions. In addition, we observed maximum effects of growth factors when a nonextremal, moderate amount of cell-fate heterogeneity exists. Our results give several insights into stochastic cell responses, including the effects of anticancer agents on cancer cells and the optimization of methods to induce the differentiation of stem cells.

## Introduction

Phenotypic heterogeneity, which has been thoroughly discussed for tumor cells, is not a unique property of cancerous cells but has also been observed in normal clonal cells in culture [1]. Differences in tumor cells have been attributed to differences in cell lineages that arise from genetic or epigenetic processes. However, even clonal cells show phenotypes that are certainly not identical because they are subject to various sources of stochasticity other than genetic heterogeneity [2]. Recent insights have suggested that molecular ‘noise’ caused by fluctuations in gene expression, signal transduction, and other processes affects phenotypic heterogeneity in organisms ranging from microbes to mammals [3]. In the presence of such noise, even cells with the same overall phenotypic profile fluctuate randomly, causing them to have subtly different phenotypes at any particular time. This is a key mechanism that generates the cellular diversity that is sometimes used as bet-hedging of bacterial persistence in *Escherichia coli* or competence in *Bacillus subtilis* [4], [5]. In mammals, several types of cells use stochastic decision-making to regulate development [6]–[10].

Although heterogeneity was observed in several types of cells, the effects of extracellular stimuli on those heterogeneous populations have not been fully clarified. Here, we used PC12 cells to attempt to address this issue. The rat pheochromocytoma clone PC12, which was developed from an adrenal medullary tumor derived from the adrenergic neural crest [11], has been used as a model of neural differentiation. Healthy PC12 cells can be grown under appropriate content percentage of serum in a medium for culture, and they have several properties that resemble those of adrenal medullary chromaffin cells [12]. In the presence of nerve growth factor (NGF), PC12 cells stop dividing, display electrical excitability, produce neurite-like outgrowths, and differentiate into cells with a sympathetic neuron-like phenotype. Although the removal of NGF for sympathetic neurons leads to cell death, the differentiation of PC12 cells appears to be reversible insofar as removal of NGF causes them to lose the properties acquired after differentiation. It has been reported that the effects of NGF on differentiation are efficient under serum-starved conditions [13]. The epidermal growth factor (EGF) receptor is also expressed in PC12 cells [12]. As in other cell types [14], EGF acts as a mitogen in PC12 cells [15]. However, the effect of EGF can be masked by culture conditions, especially the content percentage of serum [15].

The response of PC12 cells to growth factors is heterogeneous on the level of individual cells and is affected by the surrounding serum concentration, but the relationship between cell heterogeneity and cell responsiveness to growth factors has not been measured quantitatively. Here, we report the effects of the growth factors EGF and NGF under three different serum conditions, in which PC12 cells show different degrees of heterogeneity in their fate decisions. We measured the time courses of the numbers of cells in three states (proliferating, differentiated, and dead) and constructed a mathematical model. By definition, we regard concentrations of surrounding serum as an environmental condition, and regard cell responses as meaning changes in cell fate triggered by stimuli, such as EGF and NGF, at that particular serum concentration.

In this study, we first used entropy values to define the heterogeneity of a population of cells containing a large fraction of proliferative cells, and found that the entropy values increased as a function of time, suggesting that stochastic cell-fate decisions in single PC12 cells increased the heterogeneity of the population, regardless of the surrounding serum. This heterogeneity decreased as the concentration of the surrounding serum increased. We made a simple mathematical model assuming that cells determine their fates with constant probabilities, and estimated the probabilities for which the model can explain the experimental results, based on Bayes' theorem. This method enabled us to use experimental data collected using populations of cells to estimate the rates with which single cells made cell-fate decisions. Based on the model and the parameter values, we redefined the effects of the growth factors: EGF increased the proliferation rate at the single-cell level, although the effect could be covered and was affected by the surrounding serum at the population level, as shown in previous experiments. At some serum concentrations, EGF (NGF) even directed differentiation (proliferation) at the single-cell level. Even when efficient responses to the growth factors were observed at the population level, the growth factors only stochastically directed single cells to different cell fates. We evaluated the strength of the responses to the growth factors at the population level on a phase portrait using the Malthus coefficient and the fluxes of the three phenotypes, and examined the relationship between cell heterogeneity and cell responsiveness. The strength of the responses to EGF and NGF as a function of entropy (cell-fate heterogeneity) peaked at a moderate entropy value. Finally, we showed that the relationship between the means and variances of the local cell densities obeyed a power-law relationship, which could be explained by a stochastic simulation that supported the idea that the cells have a single set of transition rates with constant values under each condition.

## Results/Discussion

### Cell-fate decisions rate in single PC12 cells

In this section, we first report experimental results that describe dynamic changes in the number of cells at the population level. Next, we propose the use of a mathematical model to calculate transition rates at the single cell level from experimental results.

#### Heterogeneity of cellular states in cell populations

**Figure 1:**
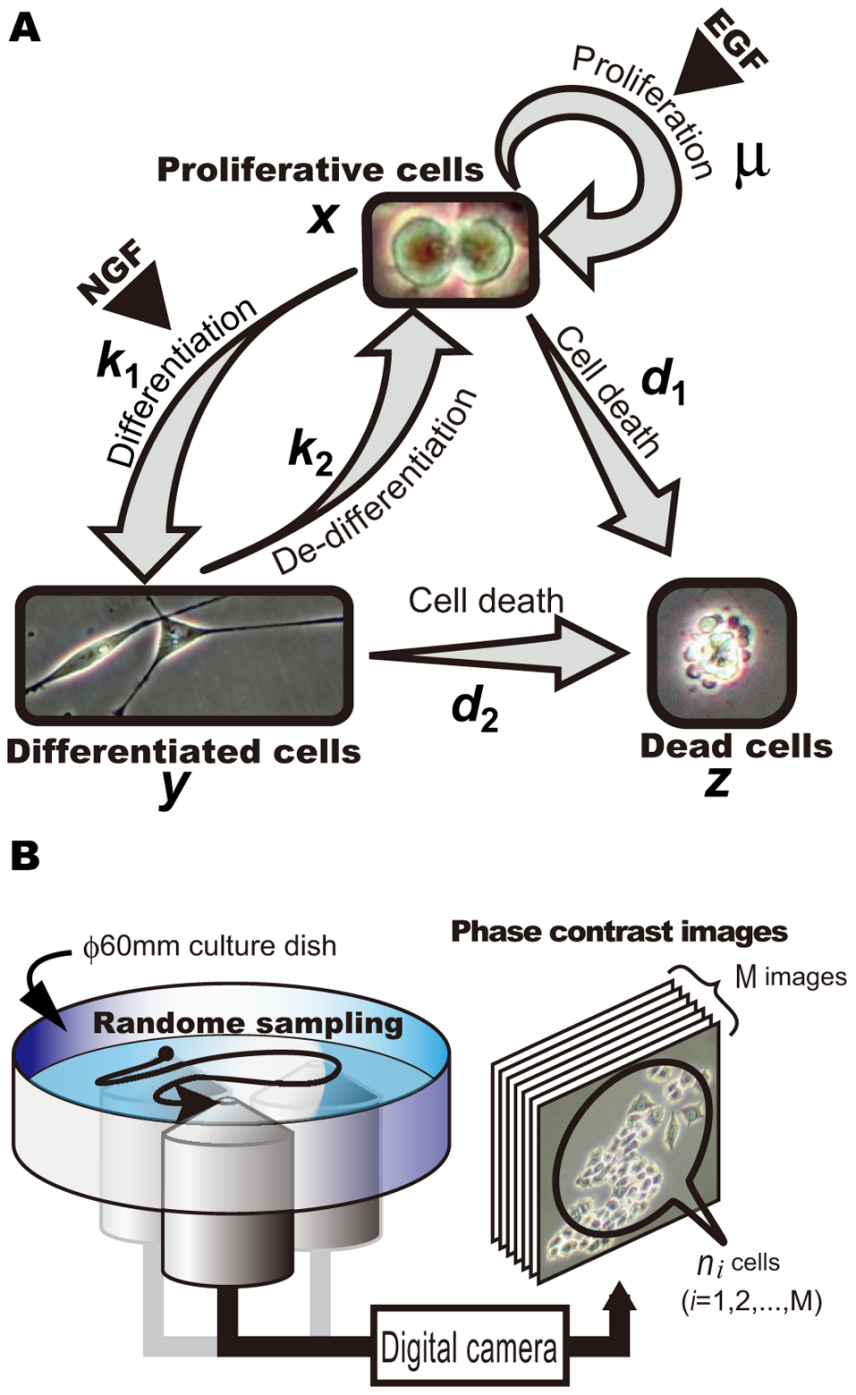
Fates of PC12 cells and experimental procedures. (A) The three typical states of PC12 cells. Whereas proliferating cells are usually rounded, differentiated cells have extended neurites, and dead cells are recognized as shrunken or fragmented cell bodies. EGF, epidermal growth factor; NGF, nerve growth factor. The values of proliferating (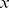), differentiated (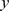), and dead (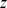) cells denote densities of each cell. Five parameters describe the transition rates of proliferation (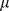), differentiation (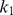), de-differentiation (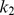), cell death from 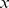 (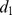), and cell death from 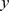 (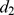). We made a mathematical model of differential equations and estimated those five parameters using Bayesian inference. Details are shown in the Models section. (B) The experimental procedures used involved photographing randomly sampled places (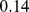 or 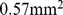) on a dish, and then counting the numbers of cells with features of each of the three states defined above. Means (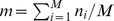) and variances (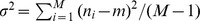) of the number of cells in each state were calculated by analysis of the entire surface of each dish.

Three typical states of PC12 cells are defined by their morphologies (Figure 1 and Materials and methods); These are (1) proliferating cells, which have a rounded shape and can potentially reproduce, differentiate, or die; (2) differentiated cells, which have neurite-like protrusions and can potentially de-differentiate or die; and (3) dead cells, which are recognized by the presence of cellular debris. The proliferating PC12 cells were passaged into culture dishes at initial densities of 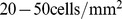. The cells did not reach confluence (over 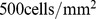) in the five days of our experimental period. We measured the mean densities of each of the three phenotypically distinct populations in culture daily in 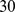 randomly selected fields in a single dish (Figure 1).

**Figure 2:**
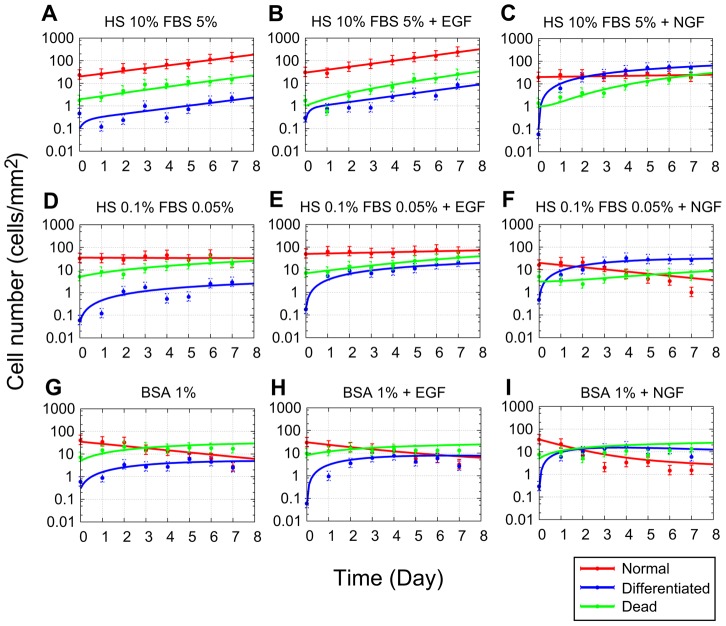
Time courses showing changes in the numbers of cells in the presence or absence of epidermal growth factor (EGF) or nerve growth factor (NGF). Time courses of three cell states were observed in experiments (circular dots), and fitted simulation results (lines) for the high serum (A–C), low serum (D–F), and serum free (G–I) conditions in the presence or absence of EGF or NGF. For each experiment, we counted 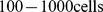 to calculate the average number of cells for each day. Typical results of four independent experiments in each condition are shown. The parameters in Table S1 were applied to simulate a mathematical model. For each figure, lines denote the average number of proliferating (red), differentiated (blue), and dead (green) cells. Error bars denote 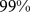 confidential region fitted by exponential distribution. HS, horse serum; FBS, fetal bovine serum.

**Figure 3:**
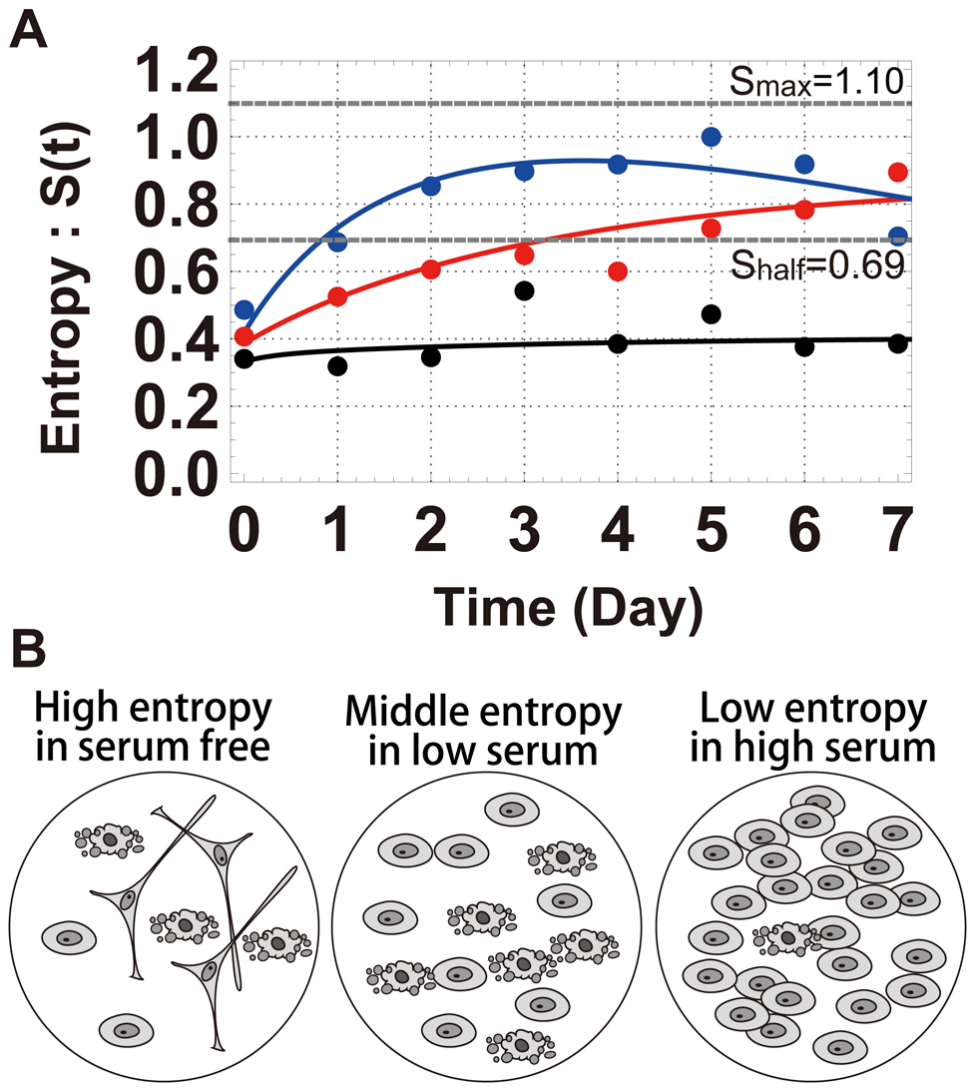
Changes in entropy with time. (A) Serum conditions used were 10% horse serum (HS) and 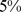 fetal bovine serum (FBS) (black), 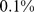 HS and 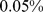 FBS (red), and 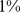 BSA (blue). The definition of entropy is 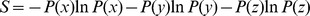. The dots show the experimental results and the lines are the results of simulation. The maximum entropy value (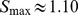) was determined for when 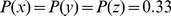, and the middle entropy value (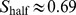) was determined for when 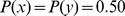 and 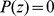. The parameter values in Table S1 were used for the simulations. (B) Under serum-free conditions, the entropy was high, and the numbers of the three cell types on the dish were similar. Under low-serum conditions, levels of entropy were intermediate, and most of the cells were either proliferating or dead. Under high-serum conditions, most of the cells were proliferating.

We compared the cell-fate processes under control conditions, in which the cells were cultured without additional growth factors but in the presence of three different concentrations of serum (Figures 2A, D, and G). In the presence of high serum concentrations (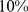 horse serum (HS) and 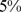 fatal bovine serum (FBS); which is the normal serum concentration used to culture PC12 cells) proliferated efficiently (approximately 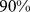 of cells were proliferating) and grew exponentially. However, even in the presence of a high serum concentration, the number of cells that differentiated or died also increased, maintaining constant fractions (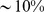). In the presence of a low serum concentration (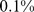 HS, 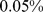 FBS), the number of proliferating cells was almost constant, and the number and fraction of differentiated or dead cells increased. Under the serum-free condition (culture medium containing 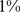 bovine serum albumin (BSA)), the number of proliferating cells decreased, whereas the numbers of dead and differentiated cells increased. Specifically, the fraction of differentiated cells increased 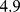-fold or 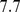-fold following growth under low-serum conditions or serum-free conditions, respectively, compared with the fraction under high-serum conditions. Therefore, even under the control conditions, a population of cells became heterogeneous with time under low-serum and serum-free conditions. To explicitly evaluate the extent to which cell fates within a population become heterogeneous, we introduced Shannon entropy (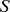), and calculated the time-dependent entropy 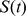 in Figure 3A.
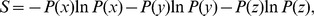
where 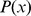, 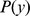, and 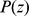 are the fractions of cells of each cell fate, and 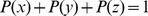. The maximum value of entropy is 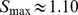 for 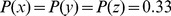, the middle value is 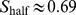 for 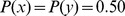 and 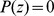, for example, and the minimum value is 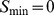 for 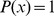 and 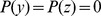, for example. When the value of entropy is 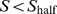, the heterogeneity of a population is small and the cell population largely follows a single fate. In contrast, if the fate decision of individual cells is random, the cells become heterogeneous and the value of entropy increases to 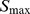. In Figure 3A, we show the entropy values as a function of time for the three control-serum conditions. At the high serum concentration, entropy was sustained at a low value of approximately 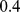 because a large fraction of cells were proliferating. Under serum-free conditions, a rapid increase in entropy was observed, and the entropy approached the maximum value (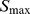), exceeding its middle value (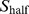), indicating the high heterogeneity of the cell population. In the presence of a low serum concentration, a moderate increase in entropy was observed, and the value was close to 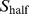. Therefore, cell heterogeneity depended on the concentration of the surrounding serum and increased as the serum concentration decreased. Cell-fate decisions displayed the greatest uncertainty under serum-free conditions, in which cells not only died but also differentiated to generate a heterogeneous population. Even at high serum concentrations, a small number of cells differentiated or died.

The growth factor EGF was added to these control conditions to evaluate how cell-fate decisions change in response to this stimulus (Figure 2B, E, and H). A saturated concentration of EGF (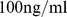) was used. Although EGF is known to induce cell proliferation, the number of proliferating cells did not explicitly increase in the high-serum concentration in the presence of EGF when compared with the control condition. In the presence of low-serum concentrations, slight increases in the number of proliferating cells were detected in the presence of EGF, but differentiation was also clearly accelerated. Under serum-free conditions, proliferating cells decreased even in the presence of EGF. Therefore, the responses of cells to the EGF stimulus depended on the concentration of surrounding serum. This suggests that EGF not only affects the proliferation of cells, but also affects their differentiation, although the effects of EGF on cell death were obscure for all of the serum conditions tested.

When we added the growth factor NGF, differentiation was accelerated under all of the serum conditions tested (Figure 2C, F, and I). A saturated concentration of NGF (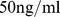) was used. Under high-serum conditions, the number of proliferating cells was sustained for a week in the presence of NGF, whereas it increased under control conditions. Thus, both the number and proportion of differentiated cells increased. NGF did not completely suppress cell death, as shown by the increased number of dead cells. However, under the low-serum condition, the number of proliferating cells decreased as the number of differentiated cells increased. In contrast, the number of proliferating cells remained constant with a slight increase of the number of differentiated cells under control conditions. The sustained composition of the surviving cells resulted from NGF-mediated stimulation of the transition of the proliferating cells into differentiated cells. Under serum-free conditions, changes in the numbers of proliferating cells over time were similar in the presence or absence of NGF, but the number of differentiated cells was larger in the presence of NGF than in its absence. This result indicates that the number of surviving cells gradually decreases upon exposure to NGF, which increases the proportion of differentiated cells.

Therefore, in our experiments, cell death and differentiation were not completely suppressed under conditions that support logarithmic growth, and cells differentiate without any growth factors even under serum-free conditions. Thus, the clonal PC12 cells became a heterogeneous population under each of the serum conditions tested. In addition, the effects of EGF and NGF depended on the environmental serum conditions, with both of these growth factors affecting multiple transition pathways. To further validate this issue, we generated a mathematical model to capture these stochastic state transitions of PC12 cells. This model is discussed in the section that follows.

#### Single-cell-level state transition rates derived from a mathematical model

It is difficult to evaluate and compare the processes that determine the fates of single cells quantitatively among different conditions directly from monitoring changes in the number of cells over time. For quantification, we constructed a mathematical model that uses ordinary differential equations to capture the state transitions of cells (Figure 1A and Models section). We assumed that the observed heterogeneity of a population is caused by the stochastic fate decisions of single cells. In this model, transition rates describe the probabilities that the phenotypes of cells may change. We estimated the values of the transition rates in the experimental data based on Bayes' theorem and evaluated the effects of serum and growth factors. Estimated results based on the experiments in Figure 2 are shown in Table S1, and time courses of this model using these parameters successfully fitted most of the experimental results (Figure 2). Therefore, our state transition model can explain our experimental results by modifying parameter values of the model, suggesting that the structure of the model is valid where we did not consider cell–cell interactions.

**Figure 4:**
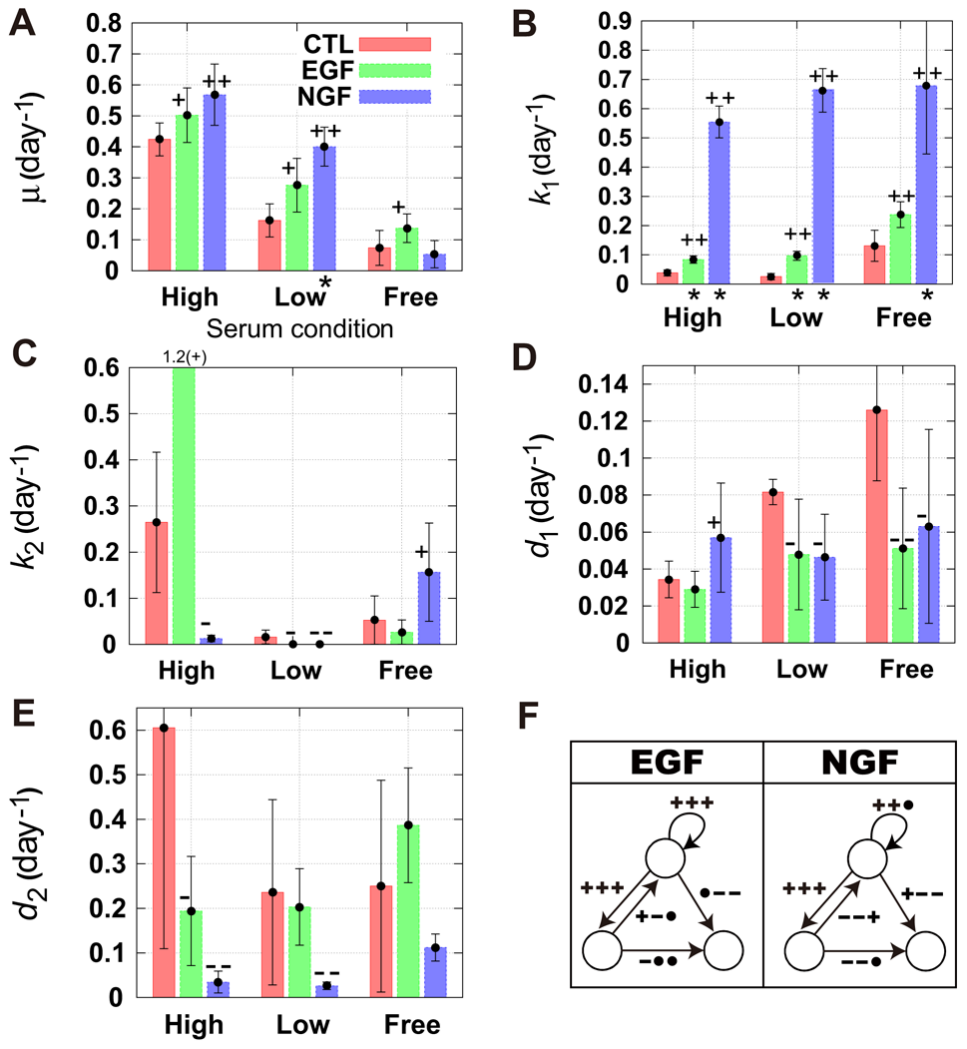
Mean estimated parameters for several serum or growth factor conditions. (A–E) Individual experiments were repeated four times (means and standard errors are shown). We applied a simple one-sided 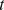
–test with reference to a serum condition of 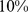 HS and 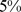 FBS, and calculated 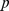
–values. Asterisks denote 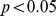. In addition, we marked ‘
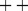
’ or ‘
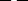
’ on the bars for the effect size 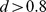, and ‘
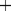
’ or ‘
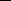
’ for 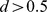 (the definition of 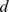 is shown in the Materials and Methods section). The plus (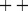, 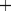) or minus (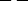, 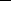) marks denote increase or decrease of mean parameter values compared with the control conditions, respectively. Details are shown in the Materials and methods section. (F) Diagrams of the responses to epidermal growth factor (EGF) and nerve growth factor (NGF) for each parameter are shown. The three sequential marks (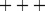, and so on) are for the high serum (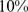 horse serum (HS) and 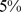 fetal bovine serum (FBS)) the low serum (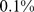 HS and 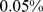 FBS), and the serum free (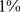 bovine serum albumin) conditions, respectively. The mark ‘
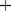
’ (‘
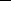
’) indicates increase (decrease) compared with the control conditions, and ‘
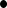
’ denotes the absence of statistically significant differences between the parameter values.

We carried out four independent sets of experiments and predicted parameter values for each experiment. The mean parameter values enabled us to estimate typical characteristics of cell-fate decisions in single PC12 cells. State transition rates at the single-cell level were compared among control conditions (Figure 4 and Figure S1). In the high-serum concentration, we can easily estimate the doubling time 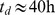 from the proliferation rate 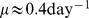. Even if cells differentiate, they immediately de-differentiate or die, with slow inductions of differentiation and death, resulting in a low entropy value (Figure 3). At the low serum concentration, the proliferation occurs with slow induction of differentiation and comparatively fast induction of death, which results in an intermediate entropy level (Figure 3). Under serum-free conditions, cells slowly proliferate with inducing differentiation and death, which results in a high entropy value (Figure 3). Each parameter had a nonzero value, regardless of the environmental serum conditions, and the values for entropy gradually increased under low-serum and serum-free conditions.

In Figure 4A–E, we show the parameter values for three environmental serum conditions in the presence or absence of either EGF or NGF. Both growth factors affected several transition pathways. For example, they drove both proliferation and differentiation, whereas for other transitions their effects depended on the environmental serum conditions. Whereas EGF increased the proliferation rate 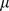 for all serum conditions, NGF increased the differentiation rate 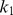 for all serum conditions. However, we found that NGF increased the proliferation rate 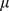 in the presence of serum, and the differentiation rate 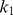 was increased affected by EGF for all serum conditions at the single-cell level. It has been suggested that although EGF is a mitogen for PC12 cells, and is capable of moderate stimulation of the proliferation of PC12 cells, these effects might be masked by serum conditions used for cell culture [15]. At the population level (Figure 2), it was unclear whether EGF affected cellular proliferation of cells. However, a mathematical model enabled us to derive transition rates at the single-cell level and to show that EGF stimulated the proliferation rate for all serum conditions, with the magnitude of the increase in proliferation with respect to the rates of proliferation under control conditions increasing as the concentration of serum decreased, as reported previously [15].

It has usually been suggested that NGF has an antimitogenic effect on PC12 cells [15], [16], although NGF has a mitogenic effect on cells purified from late-passage cultures of lines derived from PC12 cells [16]. Analysis of cell numbers failed to reveal any mitogenic activity of NGF (Figure 2), although the proliferation rate 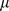 was higher in the presence of NGF compared with control conditions in high and low serum levels. A model enabled us to calculate the proliferation rate in order to distinguish proliferating cells from differentiated cells that cannot proliferate. This indicates efficient proliferation of PC12 cells in certain conditions with NGF, compared with control conditions, although large rates of differentiation mask the stimulation of proliferation in cell populations.

We also found that EGF stimulated the differentiation rate 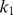 in addition to its mitogenic activity. Previous work [17] revealed that EGF triggered neuronal differentiation of PC12 cells when EGF receptors were overexpressed. In our work, we did not perform genetic manipulations. It seems that stimulation of differentiation is an intrinsic property of PC12 in response to EGF, and that its strength depends on the level of expression of the EGF receptor.

The responses of the de-differentiation rate 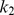 to growth factors are not yet well established. In our research, 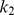 increased in the presence of EGF at the high serum concentration tested. This suggests that the number of proliferating cells increased efficiently even when differentiation occurs. In the presence of NGF, 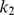 decreased compared to the control condition, and the differentiated cells efficiently increased. At the low serum concentration tested, both EGF and NGF decreased the de-differentiation rate 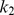, but the 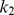 values for both EGF and NGF (including the control condition) were very small and had minimal effect on decisions related to cell fate. For the serum free condition, the de-differentiation rate 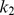 increased in a presence of NGF, suggesting inefficient differentiation. These results indicate that the effects of EGF and NGF on rates of de-differentiation depend on the serum concentration.

Suppression of cell death by NGF in serum-free medium at the cell-population level was reported previously [18]. Our approach enabled us to estimate single-cell-level responses, and we could calculate the two distinct cell death rates of proliferating and differentiated cells which could not be separated without using a mathematical model. Under high-serum and serum-free conditions, the death rate of proliferating cells 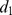 from the proliferating cells decreased when EGF or NGF were added. Nonetheless, in general, the reagents had minimal effects on the death rate of differentiated cells 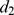. In contrast, when the serum concentration was high, NGF increased the death rate 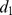, and both NGF and (to a lesser extent) EGF decreased the death rate 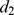. Therefore, we found that the suppression of cell death depended on serum conditions. Under serum-starved conditions, the growth factors EGF and NGF suppressed the death of proliferating cells, and under high-serum conditions, they suppressed the death of differentiated cells. The suppression of cell death was the combined effect of these two pathways, and these findings could not be achieved in the previous population level experiments that did not use mathematical models [18].

Furthermore, using a mathematical model of differential equations, 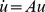 in equation (4), we can intuitively capture the time courses of the numbers of cells 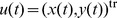 in a phase portrait (Figure S2). The number of dead cells 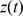 does not explicitly affect the number of cells 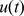, but it is implicitly included in the rate constants 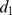 and 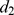. According to the simple assumptions of our model, cells under any initial set of conditions 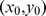 converge to the origin or diverge to infinity along one of the eigenvectors. We calculated phase portraits from individual parameter sets to understand the global features of the population dynamics. To validate the characteristics of the model, we performed an experiment to confirm that the cell-fate dynamics depend on the initial conditions (Figure S3). As predicted by the model, a number of cells moved along the vector fields in the phase plane, and finally converged to one of the eigenvectors. This result supports our assumptions that cell–cell interactions do not dramatically influence the cell-fate dynamics.

### Relationships between heterogeneity and responses to growth factors at the population level

The parameter values estimated in the previous section denote single-cell-level transition rates. However, at the population level, the effects of these parameters are very complex (see Models section), and it is difficult to evaluate the effects of the growth factors on the population dynamics from the estimated parameter values. In this section, we introduce a method to use a phase portrait to capture dynamic changes in the number of cells at the population level, and to compare the responses to growth factors under three serum conditions. We use this portrait to quantitatively characterize the responses to growth factors. Finally, we evaluate the relationships between the heterogeneity of a population and the response indexes.

#### Population-level validity of a state-transition model

**Figure 5:**
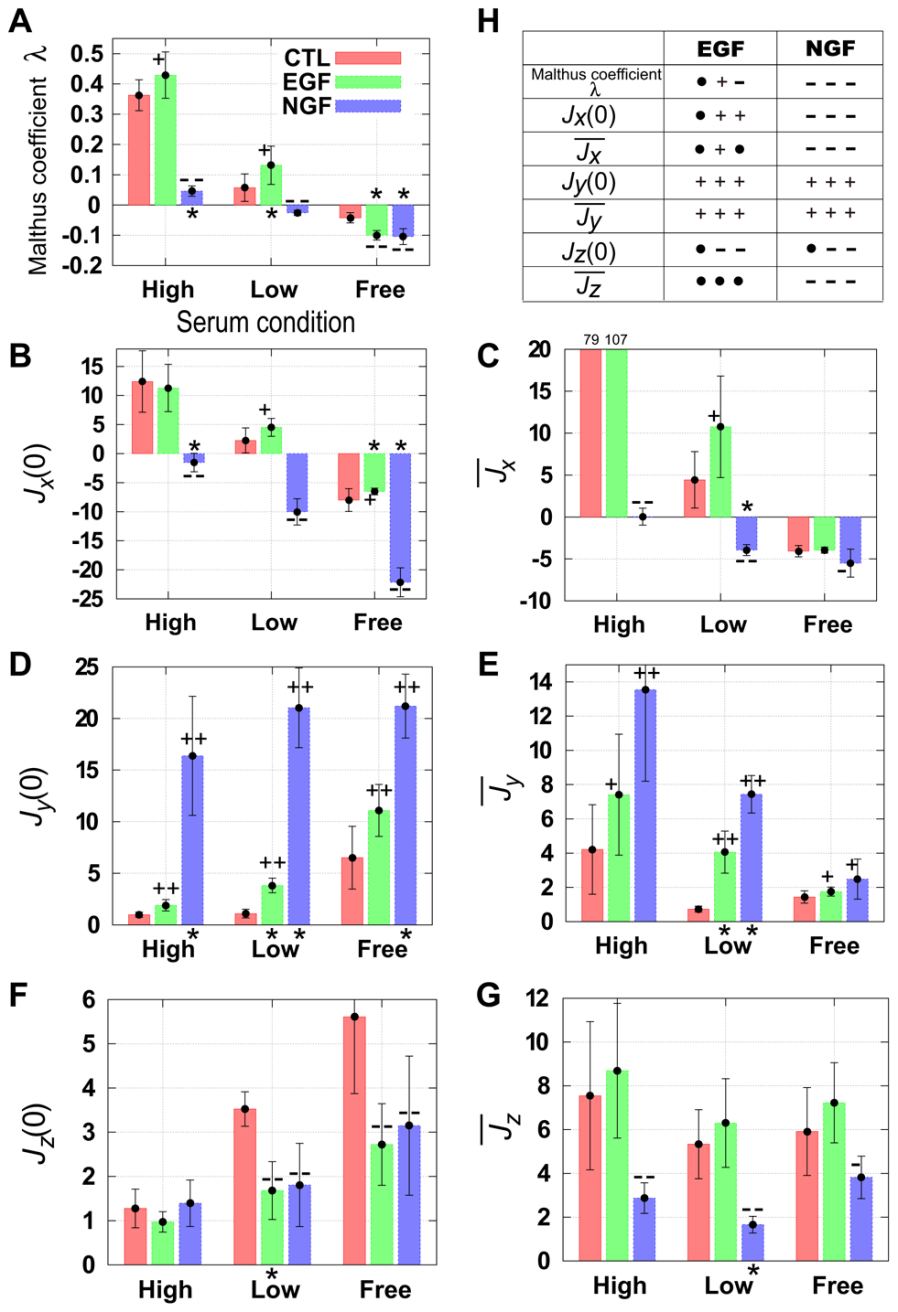
Malthusian coefficient (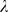) and fluxes (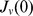 and 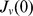) calculated from phase portraits. Malthusian coefficient (A) and fluxes (B–G) were calculated using estimated parameters in Table S1. Calculations were made in the presence or absence of growth factors for three environmental serum conditions (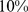 horse serum (HS) and 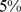 fetal bovine serum (FBS), 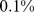 HS and 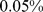 FBS, and 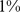 BSA). Fluxes were expressed as 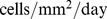. The symbols ‘
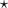
’, ‘
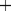
’, ‘
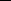
’, ‘
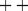
’, and ‘
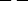
’ denote the same meanings as those defined in Figure 4. (H) Diagrams of the responses to epidermal growth factor (EGF) and nerve growth factor (NGF) for Malthusian coefficient and fluxes are shown. Three sequential marks (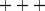, and so on) denote the same meanings of Figure 4F. The means of four independent experiments are shown with their standard errors.

We now introduce some indexes to quantify the direction of cell fate decision processes derived from a mathematical model. In the following section, we evaluate the extent of responses to growth factors compared with the control conditions that use these indexes. We propose three indices. The first is the Malthusian coefficient 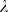, which denotes an asymptotic convergence (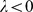) or divergence (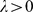) speed of change in the number of cells (details are shown in Models section). In Figure 5A, we show the calculated values of the Malthus coefficients. As indicated by the result of the previous section, a positive effect of a growth factor EGF was evident in the low serum condition. In the presence of NGF, the Malthus coefficients 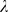 decreased compared with the control conditions for all serum concentrations. Only when the differentiation rate 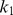 and the proliferation rate 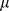 are balanced in addition to 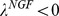 will efficient differentiation occur (Figure S2C, F, and I). Thus, consideration of the asymptotic growth rates and the results in the previous section suggests that the growth factors EGF and NGF efficiently affect decision processes that affect cell fate at a low serum concentration.

As the second and the third indices, we used the initial flux 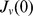 (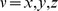) (see equation (9) in the Models section) and the mean flux 
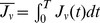
 (where 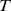 denotes an upper limit of calculating the mean flux) of cell fate processes (Figures 5B–G). The unit of those fluxes is cells/mm^2^/day. These two indices expressed similar results in response to growth factors. For the proliferating cells, EGF had a positive effect on both fluxes of cell fate decision processes 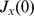 and 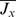 compared with the control condition only at the low serum concentration. In the high serum and the serum-free conditions, the apparent effects of EGF were not detected. In contrast, NGF had a negative effect on both fluxes for all serum concentrations. Whereas the effect of EGF depended on the environmental conditions, that of NGF was independent of the environmental conditions. For the differentiated cells, the fluxes 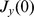 and 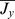 increased in the presence of both growth factors compared with control conditions. Therefore, EGF can become a differentiation factor at a population level, which is consistent with the increase in the parameter 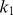 in the presence of EGF, although the positive effects of NGF on the fluxes 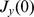 and 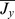 were larger than those of EGF for all serum conditions (Figure 4B). For the dead cells, the flux 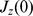 decreased under the serum-free and the low-serum conditions in the presence of EGF or NGF, and the mean flux 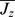 decreased in the presence of NGF.

The effects of growth factors are summarized in Figure 5H. Whereas NGF reduced the flux of proliferating cells, it increased the flux of differentiated cells and suppressed cell death relative to that under control conditions especially under the serum-free and low-serum conditions. Not only did EGF increase the flux of proliferating cells when the serum concentration was low, but it also increased the flux of differentiated cells for all serum conditions. EGF suppressed the initial flux of cell death (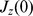) compared with control conditions as it is also suggested in the presence of NGF, but EGF did not affect the mean flux of cell death (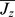). We compare the results between single and a population of PC12 cells (Figure 4F and Figure 5H). As has been suggested in ref [15], the mitogenic activity in response to EGF for a population of cells depended on concentrations of surrounding serum, and it was masked in the high-serum condition. We showed the mitogenic effects of EGF for all the serum concentrations only after we estimated parameter values of proliferating cells in single PC12 cells. In addition, we could reveal antimitogenic effects of NGF in the fluxes of a population of cells, which is consistent with the results in refs [15], [19]. We found mitogenic effects of NGF in single PC12 cells, which means that NGF promotes both proliferation and differentiation compared with the control conditions.

#### Efficient growth factor responses in a moderately heterogeneous cell population

**Figure 6:**
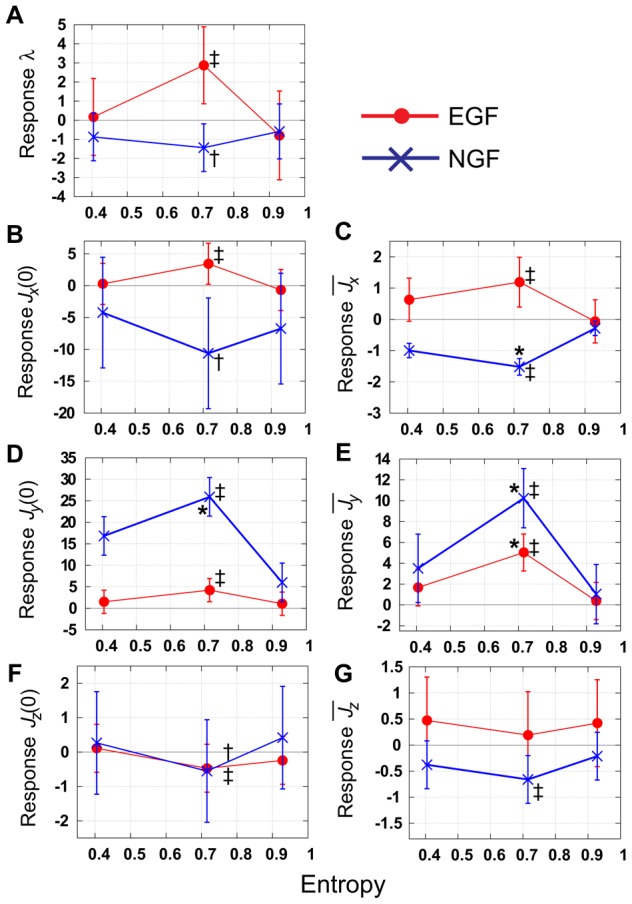
Responses to epidermal growth factor (EGF) and nerve growth factor (NGF) as functions of entropy. (A–G) Responses of the Malthusian coefficient (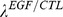 and 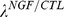), initial fluxes (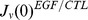 and 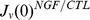), and mean fluxes (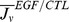 and 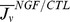) as functions of entropy. For all indexes, responses are the highest or lowest for the middle value of entropy, with many of these differences being statistically significant (asterisks denote 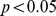, double daggers denote 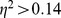, and single daggers denote 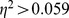 in the analysis of variances. Details are provided in the Materials and methods section. Red and blue lines denote responses to EGF and NGF, respectively.

We next compare the strength of responses to growth factors among three serum conditions (Figure 6). In order to characterize the cell fate decision processes in the environmental serum conditions, we used the entropy of cells under control conditions. We also used indices introduced in the previous section to calculate the extent of responses 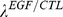, 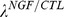, 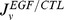, 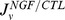, 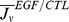, and 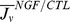 as shown in Eqs. (8), (10), and (12) in the Models section. These indices measure the extent of responses of the Malthus coefficient and fluxes to the growth factors EGF and NGF compared with the control conditions. The strength of responses to growth factors depended on the concentration of surrounding serum that affect the heterogeneity of a population. The value of the entropy with the highest response strength was approximately 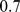 for the low serum concentration (Figure 6A–G). An explanation for this observation is that when the entropy is low, most cell fates are controlled by contained materials in serum, and additional growth factors have only limited effects. When the entropy is high, most cell fates are not controlled by serum. Instead, they are spontaneously determined, and the high level of spontaneity prevents the cells from effectively responding to external stimuli. For example, in the presence of EGF, the strength of responses indicated the maximal (Figure 6A–D, and G) or minimal (Figure 6E and F) peaks in a non-extremal, moderate entropy value for all the indices. Thus, especially for a moderate entropy value, EGF efficiently induces the proliferation of surviving cells, and not only promotes the proliferation of proliferating cells, but also induces cellular differentiation. However, cell death are most suppressed in this entropy value. In the presence of NGF, the strength of responses was maximal (Figure 6C and D) or minimal (Figure 6A, B, and E–G) for all indices; i.e., especially for a moderate entropy value, NGF efficiently induces the differentiation of cells that suppress the proliferation and death.

As shown here, we found the optimal condition for the maximal responses to the growth factors to be a nonextremal, moderate amount of cell-fate heterogeneity. An explanation of this observation is that when entropy is low, most cell fates are controlled by the materials contained in the serum, and additional growth factors have only limited effects. When entropy is high, most cell fates are not controlled by the serum. Instead, they are determined spontaneously, and the high level of spontaneity prevents the cells from effectively responding to external stimuli. The flux responses (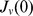, 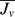) depend on the initial conditions 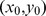. However, peaks at moderate entropy values were expected for a wide range of initial conditions (Figure S4). These types of efficient responses in the low serum concentration were also observed previously [19], with the authors of that study suggesting that PC12 cells benefited by maintaining a balance between proliferation and differentiation because the continued proliferation generated a large number of differentiated cells. The same study also showed that many genes control the balance between proliferation and differentiation. Our results indicate that the concentration of surrounding serum is also important in maintaining a balance between these two processes. Other published experimental results suggest that the concentration of serum affects control of cultured cells by the cell cycle [20]. The same study suggested that fibroblast cells grown in medium containing little serum move out of the cycle and into 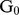 within a few hours. The 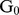 phase includes several check points when cells need some growth factors. Specifically, EGF acts during the 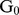 phase and supports re-entry into the 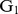 phase. Another study suggested that NGF also acts during the 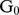 or 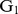 phase and induces the differentiation of PC12 cells [13]. Consideration of these results in the context of the cell cycle suggests that at a high serum concentration, many cells do not enter into 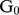 phase and proliferate efficiently, although extracellular factors have limited effects on these cells. Under serum-free conditions, many cells enter into 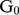 phase and extracellular factors might be anticipated to affect these cells. Nonetheless, instead the cells spontaneously differentiate, proliferate, or die without the addition of these factors. The absence of certain growth factors in serum that are needed to maintain cell cycle may diminish the effects of EGF and NGF. When the concentration of surrounding serum is low, many cells enter the 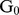 phase. This suppresses accidental cell death and spontaneous differentiation, and enables the cells to respond to extracellular factors, such as EGF and NGF. Therefore, these differences related to the cell cycle differences that depend on environmental serum conditions affect responsiveness to EGF and NGF. Responses to these growth factors are maximal when their levels are subject to moderate cell-cycle control.

### Power-law relation and validity of our model

Here, we focused on the variances of the cell densities in a culture dish. First, we show the relationships between means and variances in the experimental results, which exhibits power-law relation. Second, we extend our model in order to characterize the stochastic properties of the number of cells, and validate the assumption made in the previous sections that parameter values are constant. We also exclude the possibility that a variety of cells with distinct rate constants explain the observed variances in cell densities.

#### Experimental results exhibit power-law relation

**Figure 7:**
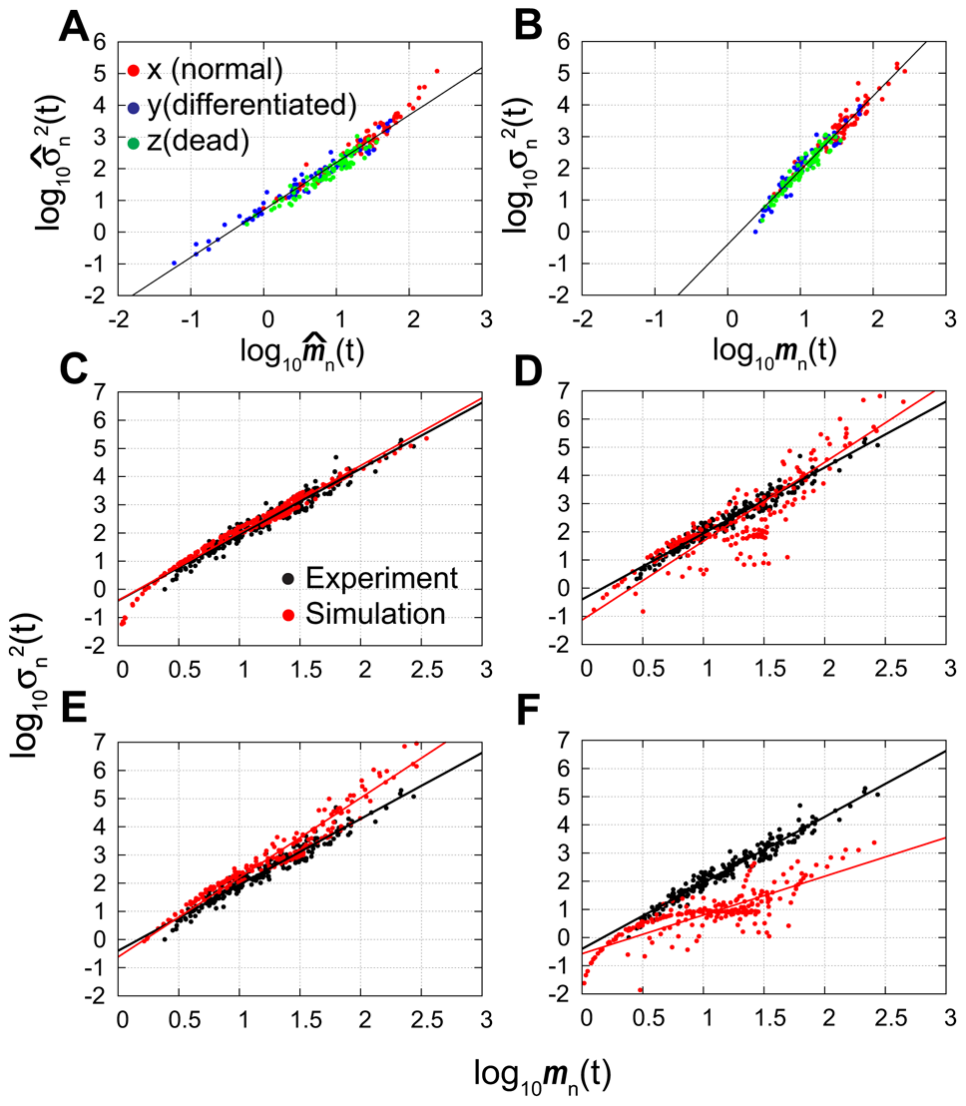
Power-law relationships between means and variances of cell density. Experimentally observed relationships between the means and variances had a slope of 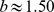 (A). When the means and variances were calculated on the basis of lognormal distribution, the slope was 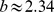 (B). Simulation results of the relationships between the means and variances with several assumptions of models were compared with experimentally determined results (C–F). (C) Model parameters were constant, and initial conditions had a lognormal distribution (model-1, 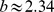). (D) Model parameters obey truncated normal distribution, and initial conditions were constant (model-2, 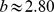). (E) Model parameters displayed truncated normal distributions, and the initial conditions displayed lognormal distributions (model-3, 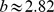). (F) Both model parameters and the initial conditions were constant (model-4, 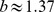). The time courses of changes in the densities of cells (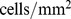) are used for experimental data. Details are shown in the main text.

To determine the distribution of cell densities, we calculated the means and the variances at time 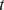; 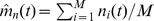, 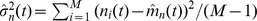, where 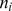 denotes the number of cells for 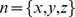, and 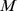 denotes the number of data at time 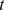 (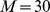 for our experiments). We found a power-law relation 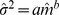 where 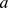 and 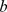 are constants (Figure 7A). Here, the experimental data shown in Figure 2 were used. We can estimate the probability distribution that 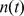 obeys by calculating the slope 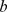 for 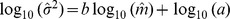. For example, when the distribution of 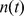 has a Poisson distribution, we have the slope 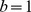, and when the distribution of 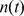 has an exponential distribution, we have the slope 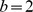. For our experiments, the slope 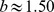 and 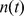 obeyed neither Poisson nor exponential distributions. This kind of simple power-law relation with several slopes 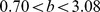 is usually observed from microorganisms to animals [21], [22], and it is suggested that 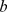 is an ‘index of aggregation’ describing an intrinsic property of the organisms from near-regular (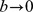), through random (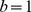) to highly aggregated (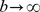). Also, the slope 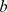 usually converges within the range 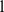 to 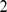, reflecting the balance between birth, death, immigration, and emigration rates [23]. In PC12 cells, immigration and emigration are small on culture dishes because of the slow migration speed compared with the observation time. Thus the slope 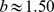 can reflect the balance between birth, death, and differentiation in our experiment.

Although these results were deduced simply from sample means and unbiased variances, our data include many zero values, 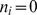, especially at time 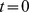 because the density of the cells was very small. We omitted zeros from our data, and we hypothesized that the data obeys a lognormal distribution (the validity of this assumption is assessed in the next section). Then the logarithm of the number of cells obey the normal distribution 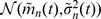, where 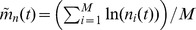, 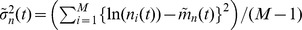. The variable 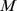 (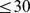) denotes the number of data with non-zero values. Therefore the means and variances of the original data (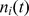) are
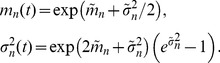
(1)The plot of 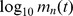 vs 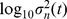 (Figure 7B) indicates a power-law relation with a slope of 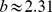 for an equation 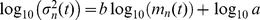. In equation (1), we can predict that the slope 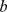 converges to 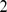 for a constant variance of 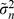 (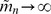). Thus the variance is not constant, but follows an equation 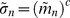 where 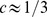, which means that the variance of the number of cells increases as the mean number of cells increases.

#### A stochastic model with constant parameters exhibits the observed power-law relation

Here, we predict the origins of the relationships between means and variances for our experiments by constructing a stochastic version of a model for the equation (3) (Models section). Even when the parameters 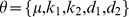 are constant, the cell-fate decision of each cell is stochastic. When we calculate the state transition process for each cell, the number of cells shows a stochastically changing trajectory. We evaluated whether constant parameters are sufficient to explain the observed variances after repeated calculation of these trajectories.

We assumed that the parameters (i-1) were constant, as defined in Table S1, or that (i-2) obeyed a truncated normal distribution, as defined in the Models section. We also assumed that the initial conditions (ii-1) were constant, as defined in Table S1, or (ii-2) obeyed a lognormal distribution. Using these simple assumptions, we made four models: model-1 with assumptions (i-1) and (ii-2), model-2 with assumptions (i-2) and (ii-1), model-3 with assumptions (i-2) and (ii-2), and model-4 with assumptions (i-1) and (ii-1). Using model-1, we could successfully achieve a power-law relationship between means 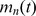 and variances 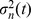 with a slope of 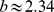, which was approximately the same as our experimental result (Figure 7C). Other models did not fit our experimental results (Figure 7D–F). Furthermore, although the power-law relation with a slope of 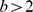 could not be explained with a simple birth–death process model with a slope 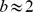 (Models section), it could be explained by our birth–death–differentiation model could, and these results did not depend on the environmental serum conditions. Thus, a stochastic model-1 corresponds to the experimental results, and the transition rates of it are constant, with the low level of noise that was assumed in the previous sections. We calculated the distribution of the number of cells using model-1 (details are shown in the Models section), and showed that the number of cells in the results of simulations usually followed a lognormal distribution (Figure S5), which supports the assumption that the distribution is lognormal under the initial conditions. The power-law relationship with 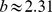 and log normal distribution were reproduced robustly in simulations using various parameter values and initial distributions (Fig. S6).

As shown here, a stochastic model with constant parameter values is sufficient to explain the observed power-law relation. Therefore, it is highly probable that a cell fate is decided by stochastic fluctuations in intracellular reactions. However, what decides the rates of fate transitions is still unknown. Interpretations of power-law relations using mathematical models have been performed in several contexts [23], [24]. Earlier work [23] suggested that a power-law relation was caused by fluctuating environmental conditions, and that population rate parameters became random variables. They focused on the density effects of populations and assumed that only a parameter of density-dependent coefficient was random. However, in the present work, our model did not include density effects or other cell–cell interactions, and it was sufficient to consider constant parameter values. Analytical predictions of the power-law relation using some birth–death processes were also suggested previously [24]. The same studies also proposed multi-species models, but did not consider complex state transition model as our model did. Our model suggests that the balance between birth, death, and differentiation affects slopes of power-law relations.

### Concluding remarks

**Figure 8:**
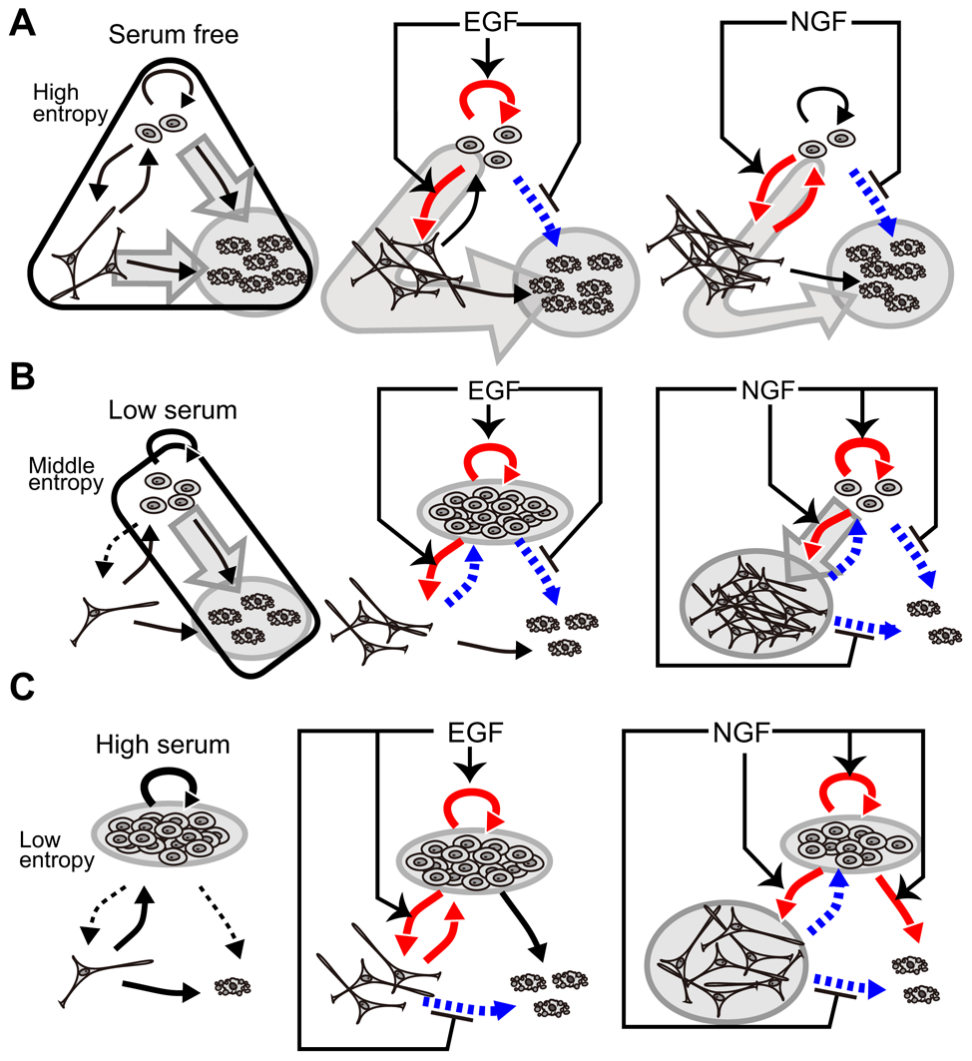
Responses to growth factors. Responses to growth factors depended on the use of no (A), low (B), and high (C) serum conditions. Under control conditions, the black thick lines denote the increase of parameter values affected by serum-containing conditions compared with the serum-free condition, and the black dotted lines denote the decrease of the same parameter values. Under each serum condition, the red thick lines denote the increase of parameter values affected by growth factors, and the blue dotted lines denote the decrease of the same parameter values. Arrows and hammer-head arrows of epidermal growth factor (EGF) and nerve growth factor (NGF) denote increase or decrease of parameter values compared with the control conditions, respectively. Gray circles and arrows indicate the population-level cell fate in each conditions.

We analyzed the time courses of the cell fate decision processes of PC12 cells after the addition of either of the growth factors EGF or NGF under three different environmental serum conditions. The results are summarized in Figure 8A–C. The population of cells became heterogeneous under all of the conditions tested, and high concentrations of serum suppressed the level of heterogeneity. The effects of growth factors depended on the environmental serum conditions, with each of the two growth factors affecting several transition pathways. Using a mathematical model, we could derive the effects of growth factors at the single-cell level. Stochastic single-cell responses to growth factors induced differentiation following exposure to EGF and proliferation following exposure to NGF. The use of phase portraits to capture dynamic changes in cell-fate decisions at the population level enabled us to evaluate the effects of serum concentrations and growth factors, and to discover conditions that promote efficient responses to growth factors. Moreover, we found that responses to growth factors were efficient when an appropriate concentration of serum induced a population with a moderate degree of heterogeneity. Finally, we have demonstrated a power-law relation between the means and variances of the local cell density. Stochastic simulation of our model could explain these results and support the validity of our model.

## Materials and Methods

### Cell culture

Rat PC12 pheochromocytoma cells from the Riken Cell Bank (Tsukuba, Japan) were cultured and maintained at 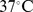, 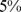 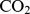 in Dulbecco's Modified Eagle Medium (DMEM, containing 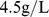 glucose) supplemented with 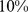 horse serum (HS) and 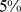 fetal bovine serum (FBS). Cells were transferred to a 60-mm culture dish with a density of 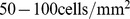. One day after the transfer, the medium was exchanged for DMEM without phenol red and supplemented with three different concentrations of serum: (i) 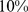 HS and 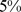 FBS (high-serum condition), (ii) 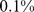 HS and 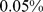 FBS (low-serum condition), and (iii) no serum but supplemented with 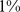 bovine serum albumin (BSA) (serum-free condition). Two days after subculture, cells were treated with 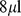 of 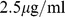 mouse 2.5S nerve growth factor (NGF) (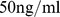 final concentration; Alomone Labs., Jerusalem, Israel), 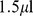 of 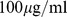 recombinant murine epidermal growth factor (EGF) (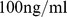 final concentration; Peprotech, London, UK), or 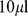 of Hank's balanced salt solution for controls. The medium and the reagents were exchanged every second day.

### Cell count

To count the densities of proliferating, differentiated, and dead cells, thirty images of living PC12 cells within thirty areas of 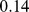 or 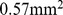 in size were taken for each dish every day after subculture using a phase-contrast microscope. The states of cells were determined from the morphologies in the captured images, with proliferating cells identified by their rounded shapes and their failure to extend neurites. The differentiated cells were defined by extension of at least one neurite with a fiber length longer than the diameter of the cell body. Dead cells were identified as shrunken or fragmented cell bodies. Examples of the micrograph of the three states of cells are shown in Figure 1A. Four independent experiments were done and the results of a typical experiment from the four are shown in Figures 2 and 7. We used the parameter set estimated from the typical experiment (Table S1) to prepare the data shown in Figure 3, and Figure S5. The average values of estimated parameters or fluxes calculated from the four independent experiments were used to prepare Figures 4, and 5.

### Statistical verification methods

In addition to using the 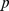-values determined using a 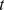-test, we used effect size 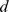 to compare two groups of data:
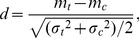
(2)where 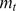 denotes a mean for treatment group and 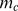 denotes a mean for control group, and 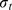 and 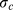 denote standard deviations for them. This value compares distance of mean values of two groups with mean values of standard deviations of them. When the value of 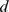 is large, the difference between two groups is large compared with the standard deviations. Significant values of effect size 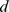 are arbitrarily defined depending on research fields, although the values of 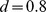 for the large effect size, 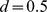 for the middle effect size, and 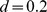 for small effect size are usually used [25].

Analysis of variance was used when more than three population averages were available for comparison. We assumed that there were 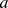 levels or conditions of experiments 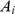 (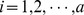) and that experiments were repeated 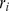 times in each level 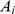. We defined the value 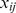 as 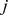
th data in a level 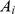. For example, a level 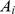 denoted each serum condition, and we repeated 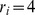 times in each level. The effect size 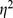 was defined as follows
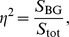
where 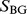 denoted the between-groups sum of squares, and 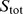 denoted the total sum of squares:
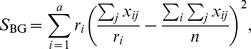


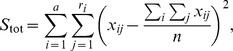
where 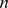 denoted the total number of experiments. The significant effect size 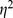 was also arbitrarily defined, but the values of 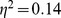 for the large effect size, 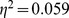 for the middle effect size, and 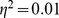 for small effect size were the typically used criteria, as in the effect size 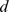 [25].

### Models

#### State transition model

To estimate the mean transition rates between the cell states, we generated a mathematical model that describes processes that decide cell fates. In this model, we assumed that (i) each cell independently determines cell fates with no cell–cell interaction, (ii) cells do not proliferate when they are differentiated, and (iii) de-differentiation occurs. The second assumption is based on reports indicating that upon treatment with NGF, cells appeared to stop dividing and the number of surviving cells was saturated [11]–[13], [15], [18].
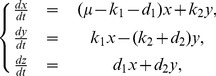
(3)where 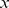, 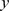, and 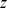 denote the mean densities (cells/mm^2^ ) of proliferating, differentiated, and dead cells, respectively. The parameters 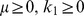 and 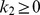 denote the proliferation, differentiation, and de-differentiation rates (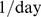), respectively. The parameters 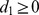 and 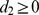 denote the death rates of the proliferating and differentiated cells (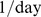), respectively. Using the limited number of experimental data 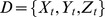 where 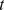 denotes the days after stimulation (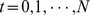), we estimated the parameter values in equation (3) applying Bayesian inference to our model (Materials and methods). A typical example of the parameter values from the four independent experimental datasets are shown in Table S1. Time courses of experimental and simulation results are shown in Figure 2. The model successfully estimated the parameters that describe the experimental results.

#### Analytical solution of a state transition model in a phase space

The velocities 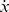 and 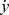 do not depend on 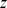, and we can simplify equations (3) to linear differential equations:
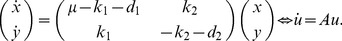
(4)The eigenvalues 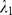 and 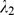, and corresponding eigenvectors 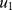 and 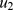 of a vector 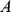 provide an analytical solution of equation (4):
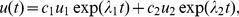
(5)where 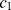 and 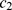 are constants depending on initial conditions 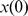 and 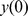. A solution 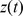 can be calculated using the equations (3) and (5). In particular, we can write down the solutions as follows:
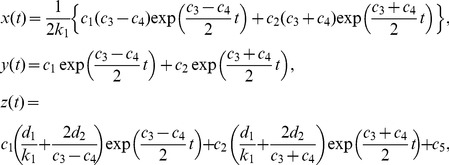
(6)where 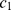, 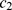, 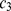, 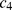, and 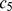 denote
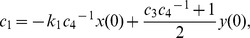


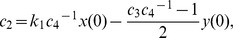


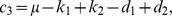


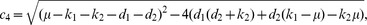


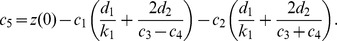
The solutions of equation (5) are categorized into several types depending on the geometrical features of trajectories around the critical point 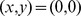 [26]. The dynamics of the number of cells are plotted on a phase space. For linear differential equations, we can simply classify the patterns of solutions depending on the parameter values of equations. When all parameters 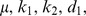 and 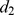 are constrained to be positive, as in the case of our model, the types of solutions are limited. In our model parameters, the solutions of the characteristic equations have two eigenvalues, and the property of the critical point is a stable node or an unstable saddle. Whereas the former means that a population of cells extincts asymptotically, the latter means that it increases infinitely. Here we define the parameters 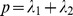, 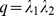, and 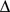, with 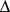 denoting a discriminant of a characteristic equation of 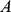. Our observation that 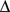 was always 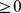 suggests that the critical point is not a center or spiral. We found that the critical point was either (i) an asymptotically stable node (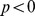 and 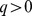), or (ii) a saddle point (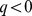). Then the vector 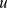 is constrained to have limited trajectories.

In our model, the parameter values are always nonnegative. Therefore, the number of cells converges asymptotically to the following equation, with a long time limit [27],
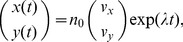
(7)where 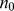 denotes an arbitrary initial condition, the Malthus coefficient 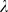 is calculated from the equation 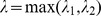, and 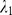 and 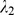 are eigenvalues of 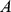 in equation (4). The corresponding eigenvector is 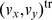. When 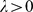, the number of total surviving cells increases, but when 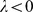, it decreases to the origin.

#### Normalized response rates in the phase space

The rates of responses to external signals compared with a control condition were defined in one of three ways: (i) the Malthusian parameter, (ii) the initial response speeds, or (iii) the time average of response speeds. The Malthusian parameter 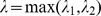, where 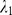 and 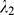 are two eigenvalues for our linear model, and the asymptotic response rates 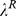 which are normalized for a control condition are defined as
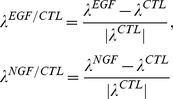
(8)where 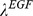 (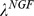) denotes the eigenvalues in the presence of growth factors EGF (NGF), and 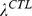 denotes those in the absence of growth factors. The initial response speeds are
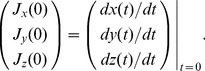
(9)Then, the normalized response speeds are
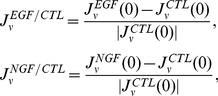
(10)where 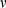 is a variable 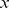, 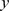, or 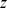, and 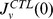, 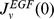, and 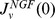 denote the net fluxes of the number of cells at time 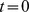 in the presence or absence of either EGF or NGF. The time averages of response speeds are
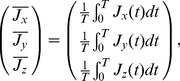
(11)where 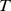 is a range of averages; here we defined 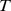 as the day at which the experiments were ended. Then, the normalized time averages of the response speeds are
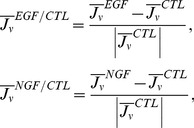
(12)where 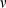 is a variable 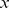, 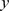, or 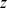. Also, 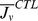, 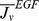, and 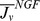 denote the time averages of the net fluxes of the number of cells at a range of time 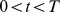 in the presence or absence of EGF or NGF.

#### Parameter estimation for a deterministic model

To estimate parameter values for our three-state model, we applied Bayesian inference to our experimental data. In addition to the differential equations (3), we assumed that the experimental data of the numbers of three-state cells, 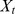, 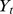, and 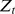, satisfy the observation equations as follows:
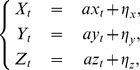
(13)where 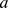 (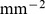) is a constant which exchanges the unit of the average density for that of the average number of cells in a microscope image. The variables 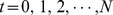 denote the observation time (day), and the parameters 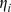 (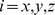) denote time-independent observation noise that satisfies an identically independent normal probability density function, 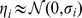. The likelihood of reproducing the experimental data 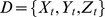 (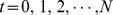), consisting of 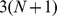 independently distributed data points with a set of parameters 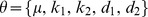 is defined as:
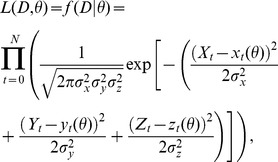
(14)where 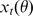, 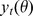, and 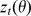 are predicted from equations (3) with parameters 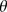 and initial conditions 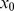, 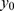, and 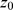. We sought to estimate a set of parameters 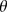, 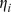, and initial conditions that maximize the above likelihood function 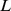. However, we only applied Bayesian inference to parameters 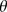. We directly estimated the initial conditions from our experimental data, which did not affect estimation of 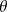. Also, we arbitrarily defined the observation noise 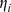 for which we could attain a sufficiently large value of likelihood 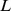. In our experiments, values of 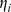 (the standard deviations 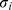 which ranges from 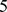 to 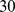) were able to adequately explain our experimental data.

The posterior probability density function 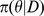 obeys the following equation based on the Bayes' theorem
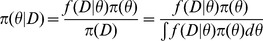
(15)

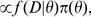
(16)where 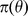 denotes the prior probability density function and 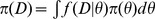 denotes the normalization constant or the marginal likelihood. A sample from the parameter posterior can be obtained using Markov Chain Monte Carlo (MCMC) sampling. Here we used the Metropolis–Hasting algorithm to produce samples from a distribution 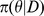. We assumed the gamma distribution as the prior distribution of 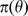, because all model parameters should have non-negative values based on biological requirements. The parameters 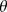 independently obey the gamma distribution as follows
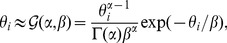
(17)where 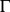 denotes the gamma function and 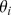 is an each component of 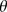. In our experimental results, possible ranges of parameters include zero and we set 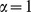 and 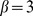, where values of 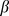 at least 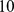 times larger did not definitely affect the values estimated with our method. Smaller values of both 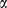 and 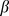 narrow the distribution, and in general, estimates become biased. Therefore, we used these values to search for parameters in a broad distribution. The candidate parameters 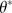 are generated using the random walk chain of a normal random number 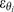 with the variance 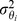:
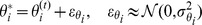
where 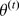 denotes a previously selected parameter set. Here we assume that the proposal distribution is symmetric
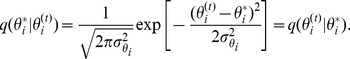
Therefore, the acceptance rate of candidate parameters is
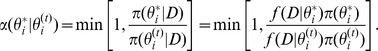
To estimate an optimal parameter set, we applied simulated annealing to our data set. Thus the acceptance rate can be modified to
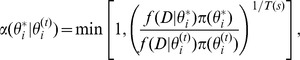
where we assumed that the function 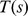 (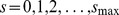) which is called the temperature gradually decreases (setting 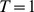 recovers Metropolis sampling) with the following four steps
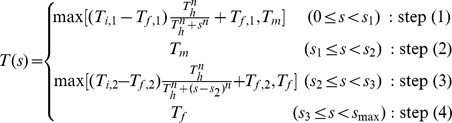
For a constant time step 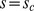 and a temperature 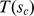, the MCMC chain was repeatedly applied for a fixed sampling number 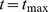. In the initial step (1), we intended to sample a wide range of parameter space, and cool down the initial high temperature 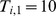 to the final one 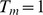 to recover Metropolis sampling. A value of 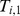 at least 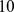 times larger did not affect the estimation of the parameter values. For a while, the metropolis sampling was performed in a second step (2) to converge the posterior parameters with distribution 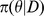. In the next step (3), we further decreased the temperature 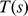 from 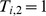 to 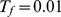 in order to estimate a mode of 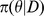. Finally, we repeated sampling with a constant temperature 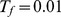 in the last step (4). In selecting the last temperature 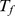, we checked the likelihood values 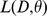 in step (4) gradually decreasing the value of 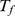. When the likelihood value was saturated, we stopped decreasing the temperature and obtained a value of 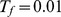. In our estimation procedures, we used the following values 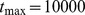, 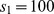, 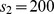, 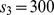, and 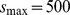. To determine a suitable parameter set for our experimental results, we used the likelihood value at a temperature 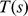; 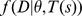. When the likelihood becomes maximum in a constant temperature 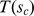, we selected a parameter set 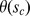 as the best for that temperature. Then we averaged over parameter sets 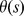 in step (4), and defined them as the estimated parameters
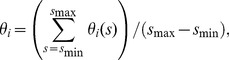
(18)where 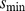 satisfies 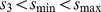 and we set 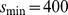 for our experiments. We monitored the selected parameter values for each step 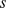, and confirmed that these numbers of steps (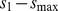) were sufficient to cause the distributions of the parameter values to converge. We evaluated the dependency of the estimation of 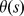 on the initial parameter values 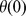, the initial temperatures 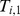, and the value of 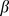 in a gamma distribution (Figure S7). In our estimation methods, at least 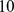 times large values of 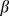 and 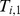, and four orders of different initial parameter values did not affect estimation of parameter values.

#### A stochastic version of a state transition model

Here, we seek to make a stochastic model. A scheme of cell fate decision processes is as follows
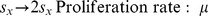


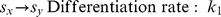


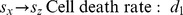


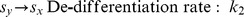


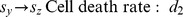
where 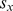, 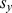, and 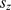 denote states of each cell. All events are first order processes, and rate constants for differential equations can be applied directly to these reaction schemes. We calculated this scheme using the Gillespie algorithm with absorbing boundary conditions at 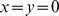 [28], and derived means 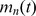 and variances 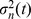 introduced in equation (1). Here, we can assume several possibilities for (i) the distributions that the parameter values obey, and (ii) the initial conditions 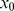, 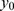, and 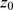 of the densities of cells. For the parameter values, we assume that (i-1) the parameters are constant as defined in Table S1, or (i-2) the parameters obey a truncated normal distribution with the probability density function of
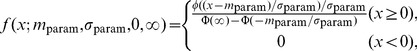
(19)where 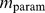 (or 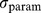) is one of parameter values 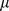, 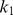, 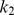, 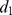, 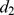, 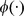 denotes the probability density function of a standard normal distribution 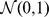, and 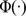 denotes the distribution function of it. For the initial conditions, we assume that (ii-1) the initial conditions are constant as defined in Table S1, or (ii-2) the initial conditions 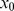, 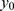, and 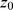 obey a lognormal distribution log 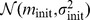 with
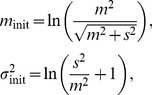
(20)where 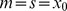, 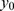, or 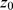.

Lognormal distributions (Figure S5) were calculated from simulation results of model-1 at day 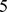, and involved 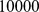 samples using parameter values in the high serum condition 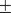 EGF and NGF. In addition, we used 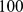-times larger values for the initial conditions in this figure than in Figure 7, and converted the values from the number of cells to the cell density. For each figure, distributions from simulations were fitted to a normal distribution 
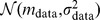
; 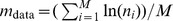 and 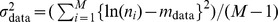, where 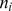 denotes the simulated non-zero density values of cells at day 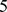 and 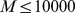.

#### Analytical solution of birth–death process

When we define the number of cells at time 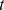 as 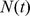, and the probability as 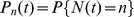, the birth–death processes are described by
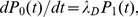
(21)

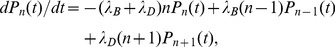
(22)where the birth rate from the sate 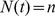 is 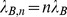, and the death rate from the sate 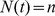 is 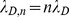. The steady state probability is obtained by
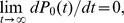


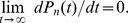
If the birth and death rates are 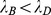, the number of cells converges to 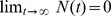 and the probability becomes 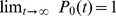, which suggests extinction of a population. If the birth and death rates are 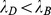, the number of cells converges to 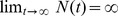 and the probabilities are 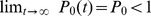 and 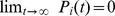 (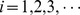); this suggests an infinite increase of a population with probability 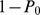. The means and the variances of the number of cells 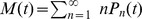 and 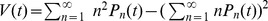 can be calculated as follows
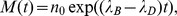


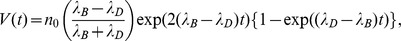
where 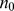 denotes the initial condition 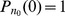 [29]. Here we define the logarithm of the mean and variance
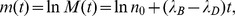


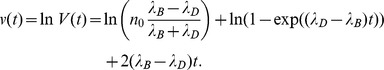
Therefore when the birth and death rates satisfy 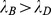, the slope becomes
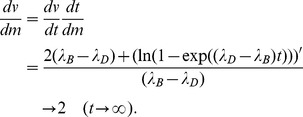



## Supporting Information

Figure S1
**Averages of the estimated parameter values for the control serum conditions from four sets of independent experiments.** We applied a simple one-sided 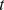
–test with reference to a serum condition of 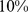 HS and 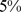 FBS, and calculated 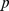
–values. Asterisks denote 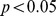. In addition, we marked ‘
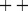
’ or ‘
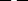
’ on the bars for the effect size 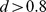, and ‘
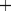
’ or ‘
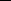
’ for 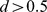. The plus (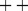, 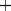) or minus (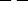, 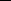) marks denote increase or decrease of mean parameter values compared with the control conditions, respectively. Details are shown in the Materials and Methods section.(TIF)

Figure S2
**Phase portraits of cell fate transitions under different serum conditions or growth factor conditions.** Phase portraits for the high serum (A–C), the low serum (D–F), and the serum free (G–I) conditions are shown. Parameters of Experiment 1 in Table S1 were used to calculate the dynamics. Two red lines in each panel denote eigenvectors. Blue dots denote experimental results and blue lines denote simulation results with parameters estimated using experimental results. Arrows are fluxes at each point in a phase. BSA, bovine serum albumin; EGF, epidermal growth factor; FBS, fetal bovine serum; HS, horse serum. The data in these figures clearly show differences in cell responses to growth factors, which depend on the concentrations of surrounding serum. For the low serum concentration, the number of cells gradually converges to the origin in the control condition, but it begins to increase in the presence of EGF. Furthermore, the number of differentiated cells increases, but the number of proliferating cells decreases in the presence of NGF. Approximately, 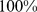 (determined as 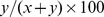) of cells are differentiated when the number of proliferating cells converges to 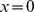, and the fraction is sustained for several days until the number of differentiated cells becomes 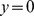. At the high serum concentration, we cannot find definite effects of EGF addition on the number of cells 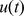. After the addition of NGF, the number of differentiated cells 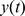 increases with the accumulation of proliferating cells 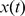, indicating an inefficient differentiation. Under the serum-free condition, the number of cells converges to the origin for the three cases, and growth factors affect the extent of differentiation especially in the early stages of cell-fate processes (immediately after addition of growth factors).(TIF)

Figure S3
**Dependency of the initial conditions on the dynamics of the number of cells in a phase portrait.** (A) The dynamics of the number of cells under the low serum condition (HS 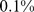 and FBS 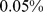) in the presence of NGF (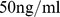). We added NGF at Day 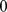, and cultured for the first fix days (Day 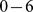 in blue-solid lines). The number of differentiated cells efficiently increases. Blue-dashed lines are for Day 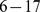. (B) We cultured cells in the low serum condition without NGF for eleven days (Day 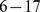 in blue-solid lines). Blue-dashed lines are for Day 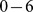. At Day 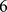, we washed out the medium containing NGF, and refreshed medium. The number of differentiated cells drastically decreases, and the number of proliferating cells increases along the one of eigenvectors. For both figures, phase portraits of experiment 3 in Table S1 were used. Blue-dashed lines are only for indication.(TIF)

Figure S4
**Dependency of response rates on initial conditions.** The initial response speeds 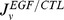 and 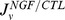 (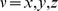) (A), or the time averages of response speeds 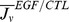 and 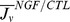 (B) for several initial conditions (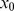 and 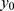) and serum conditions (High serum: 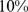 horse serum (HS) and 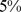 fetal bovine serum (FBS); low serum: 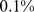 HS and 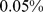 FBS; serum free: 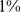 bovine serum albumin.) were calculated. The initial condition 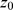 do not affect those speeds. For each initial condition, we compared the speeds among three serum conditions. When the speed was maximal (minimal) in the middle entropy condition, we plotted a red (blue) point on a graph, respectively. When the speeds monotonically changed, the region of a graph is white. Initial conditions for our experimental results were also plotted on a graph (a cross mark for the control conditions, a circled mark for EGF-added conditions, and a box mark for NGF-added conditions). Estimated parameter values of experiment 1 in Table S1 was used for calculating these figures.(TIF)

Figure S5
**Log-normal distributions of the simulated cell density.** Histograms of the cell densities (cells/mm^2^ ) at day 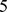 are calculated using parameters in the serum free conditions (A), the low serum conditions (B), and the high serum conditions (C) in the presence or absence of growth factors (CTL, control). Simulations have done using the parameters in the high serum condition of model-1. Black lines denote normal distribution using means and variances from simulated data.(TIF)

Figure S6
**Power-law relations under various parameter values and initial conditions.** To examine the generality of the result in Fig. 7, simulations were carried out changing the parameter values and initial distribution. (A–F) The relationship between mean and variance of cell density was calculated using parameter values 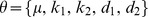 independently selected from uniform random numbers with the range of 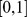. The mean initial numbers of cells 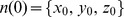 were constant 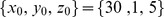 (A, C), or independently selected from 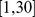 (B, D–F). Initial distribution was set to obey lognormal (A, B), exponential (C, D), or Poisson (E, F) distribution. For the initial distributions, we used the equations (20) for a lognormal distribution, a function 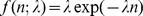 (where 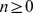) with 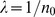 (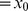, 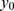, or 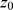) for a exponential distribution, and a function 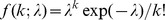 (where 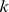 is a natural number) with 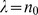 (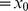, 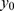, or 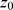) for a Poisson distribution. We simulated 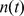 (methods were shown in models section), 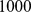 times with constant parameters and the initial number of cells to calculate means and variances at day 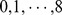 (A–E, red circles) or day 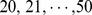 (F, red circles). We repeated 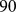 times of this procedure with randomly sampled parameters or initial conditions, and estimated the slope 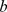 for 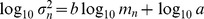 (red lines). The slopes 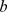 were 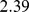 (A), 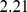 (B), 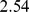 (C), 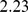 (D), 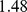 (E), and 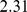 (F). The blue line in (E) denotes an equation with 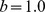 (Poisson distribution). In the figure (F), we used only 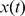 and 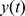, and omitted 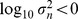 values to evaluate a slope 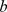. (G) Distributions of cell density were calculated at day 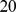 with arbitrarily defined parameter values 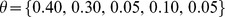 and the mean initial conditions 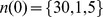 for a Poisson distribution. We simulated 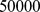 sample paths to make this distribution.As shown here, power-law relation did not depend on the specific conditions of parameter values and the initial number of cells when the initial distribution was lognormal and exponential. For Poisson distribution, the slope 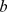 initially 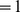 gradually increased to 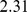 (E, F). In addition, the distribution of the number of cells became lognormal even when the initial number of cells obeyed a Poisson distribution (G). Therefore, the power-law relationship between mean and variance of cell density with 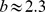 and log normal distribution of cell density after long period of cell culture seem to be general features of our three state cell fate decision model. In the simulation, cell culture started from Poisson distribution takes much longer days to reach log normal distribution than the experiments. It should be caused experimental difficulty to disperse cells completely when they transferred to subcultures.(TIF)

Figure S7
**Dependency of parameter estimations on the initial conditions.** To show the typical process of parameter estimations under different initial conditions, these figures plotted selected parameter values 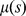 with the maximum likelihood in a constant temperature 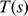 at each simulation step 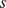. We used experimental data from experiment 2 of high serum and control condition in Table S1. The gamma distributions with parameters 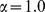, and 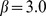 (A, C) or 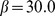 (B) were used as the prior distribution. For the initial temperature, we defined 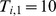 (A, B) or 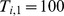 (C). We selected the final temperature as 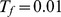 for all figures. The initial parameter values in step 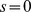 and at time 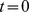 were 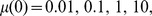 or 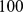, where 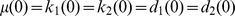. In each figure, we showed three sample paths with the same initial conditions. In all cases, a wide range of values was searched in estimation and 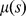 converged to similar values when 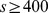. The parameter 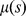 converged to 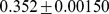 (A), 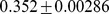 (B), and 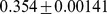 (C).(TIF)

Table S1
**Estimated parameter values in four independent experiments.** High serum: 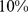 HS and 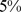 FBS, low serum: 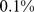 HS and 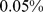 FBS, serum free: 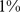 BSA. The initial conditions (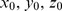) for each serum and stimulus condition did not strictly affect estimation, and we arbitrarily assigned several values which could be estimated from experimental results. Values were estimated on the basis of the results shown in Figure 2 in the main text. Estimations were done for the independent four experimental results of the time courses of the number of cells.(PDF)
