# Supplementary figures and images for: Optimality Conditions for Cell-Fate Heterogeneity That Maximize the Effects of Growth Factors in PC12 Cells
Source: PLoS Comput Biol. 2013 Nov 14;9(11):e1003320. doi: 10.1371/journal.pcbi.1003320 (PMC3828137; doi:10.1371/journal.pcbi.1003320)

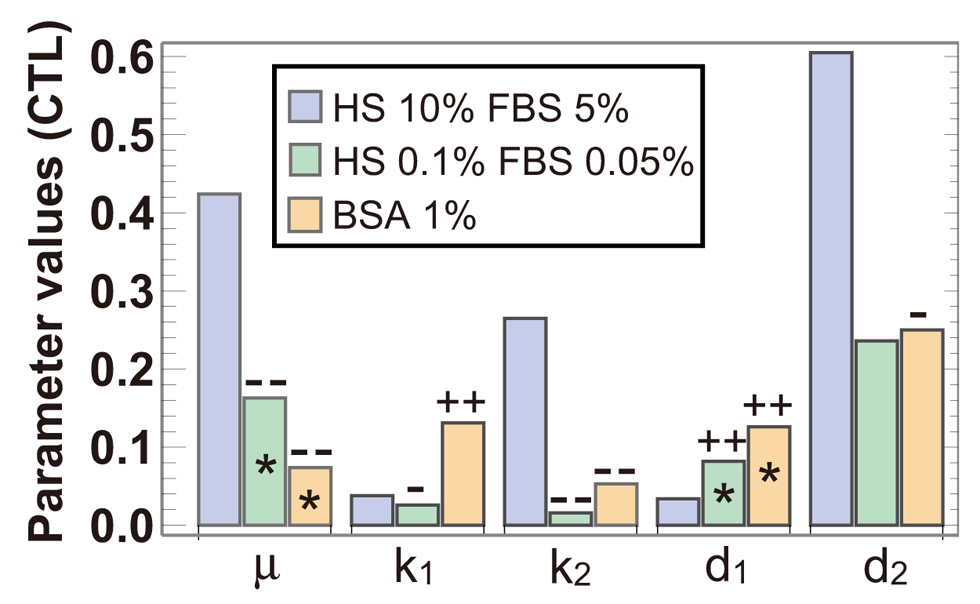

Supplement: Figure S1 — Averages of the estimated parameter values for the control serum conditions from four sets of independent experiments. We applied a simple one-sided –test with reference to a serum condition of HS and FBS, and calculated –values. Asterisks denote . In addition, we marked ‘’ or ‘’ on the bars for the effect size , and ‘’ or ‘’ for . The plus (, ) or minus (, ) marks denote increase or decrease of mean parameter values compared with the control conditions, respectively. Details are shown in the Materials and Methods section. (TIF) [file pcbi.1003320.s001.tif]

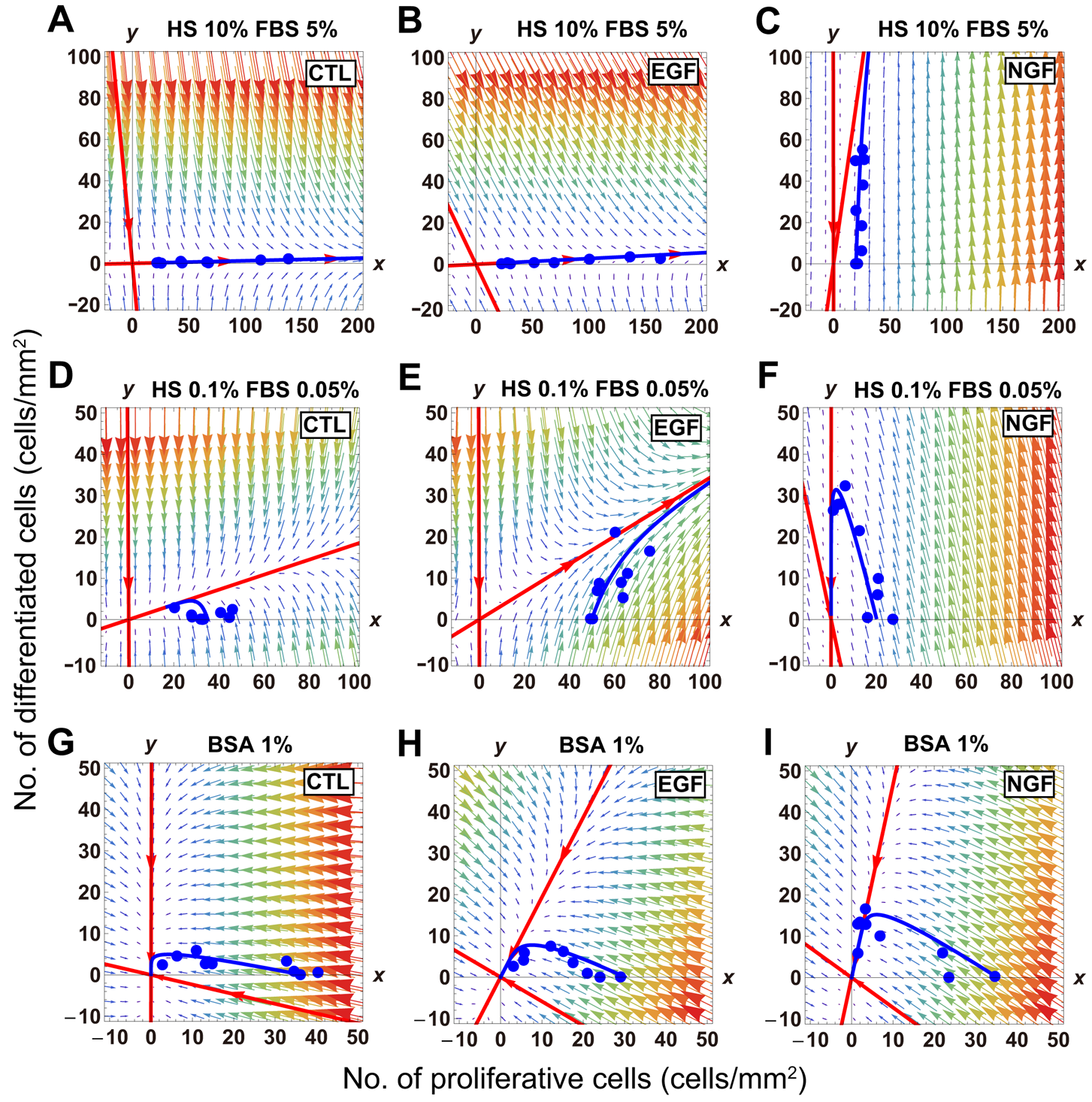

Supplement: Figure S2 — Phase portraits of cell fate transitions under different serum conditions or growth factor conditions. Phase portraits for the high serum (A–C), the low serum (D–F), and the serum free (G–I) conditions are shown. Parameters of Experiment 1 in Table S1 were used to calculate the dynamics. Two red lines in each panel denote eigenvectors. Blue dots denote experimental results and blue lines denote simulation results with parameters estimated using experimental results. Arrows are fluxes at each point in a phase. BSA, bovine serum albumin; EGF, epidermal growth factor; FBS, fetal bovine serum; HS, horse serum. The data in these figures clearly show differences in cell responses to growth factors, which depend on the concentrations of surrounding serum. For the low serum concentration, the number of cells gradually converges to the origin in the control condition, but it begins to increase in the presence of EGF. Furthermore, the number of differentiated cells increases, but the number of proliferating cells decreases in the presence of NGF. Approximately, (determined as ) of cells are differentiated when the number of proliferating cells converges to , and the fraction is sustained for several days until the number of differentiated cells becomes . At the high serum concentration, we cannot find definite effects of EGF addition on the number of cells . After the addition of NGF, the number of differentiated cells increases with the accumulation of proliferating cells , indicating an inefficient differentiation. Under the serum-free condition, the number of cells converges to the origin for the three cases, and growth factors affect the extent of differentiation especially in the early stages of cell-fate processes (immediately after addition of growth factors). (TIF) [file pcbi.1003320.s002.tif]

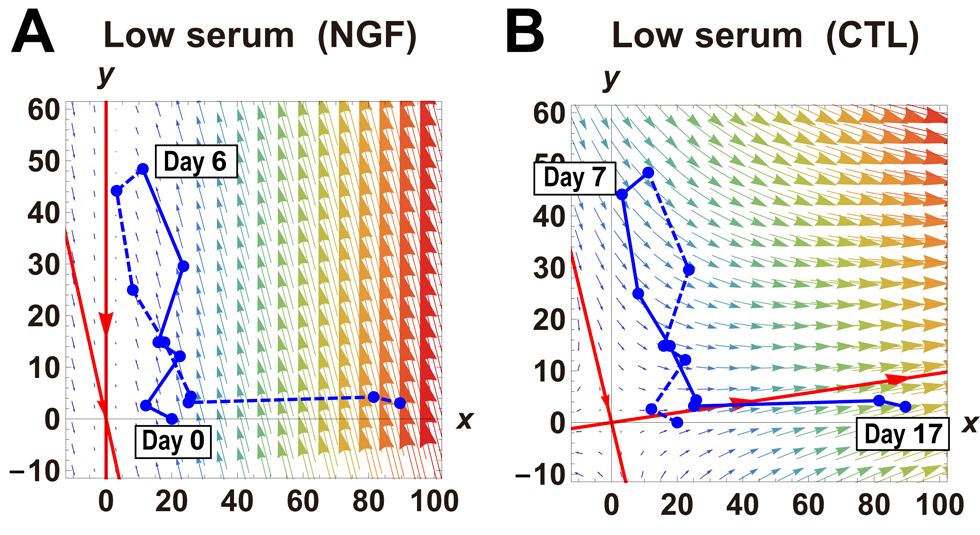

Supplement: Figure S3 — Dependency of the initial conditions on the dynamics of the number of cells in a phase portrait. (A) The dynamics of the number of cells under the low serum condition (HS and FBS ) in the presence of NGF (). We added NGF at Day , and cultured for the first fix days (Day in blue-solid lines). The number of differentiated cells efficiently increases. Blue-dashed lines are for Day . (B) We cultured cells in the low serum condition without NGF for eleven days (Day in blue-solid lines). Blue-dashed lines are for Day . At Day , we washed out the medium containing NGF, and refreshed medium. The number of differentiated cells drastically decreases, and the number of proliferating cells increases along the one of eigenvectors. For both figures, phase portraits of experiment 3 in Table S1 were used. Blue-dashed lines are only for indication. (TIF) [file pcbi.1003320.s003.tif]

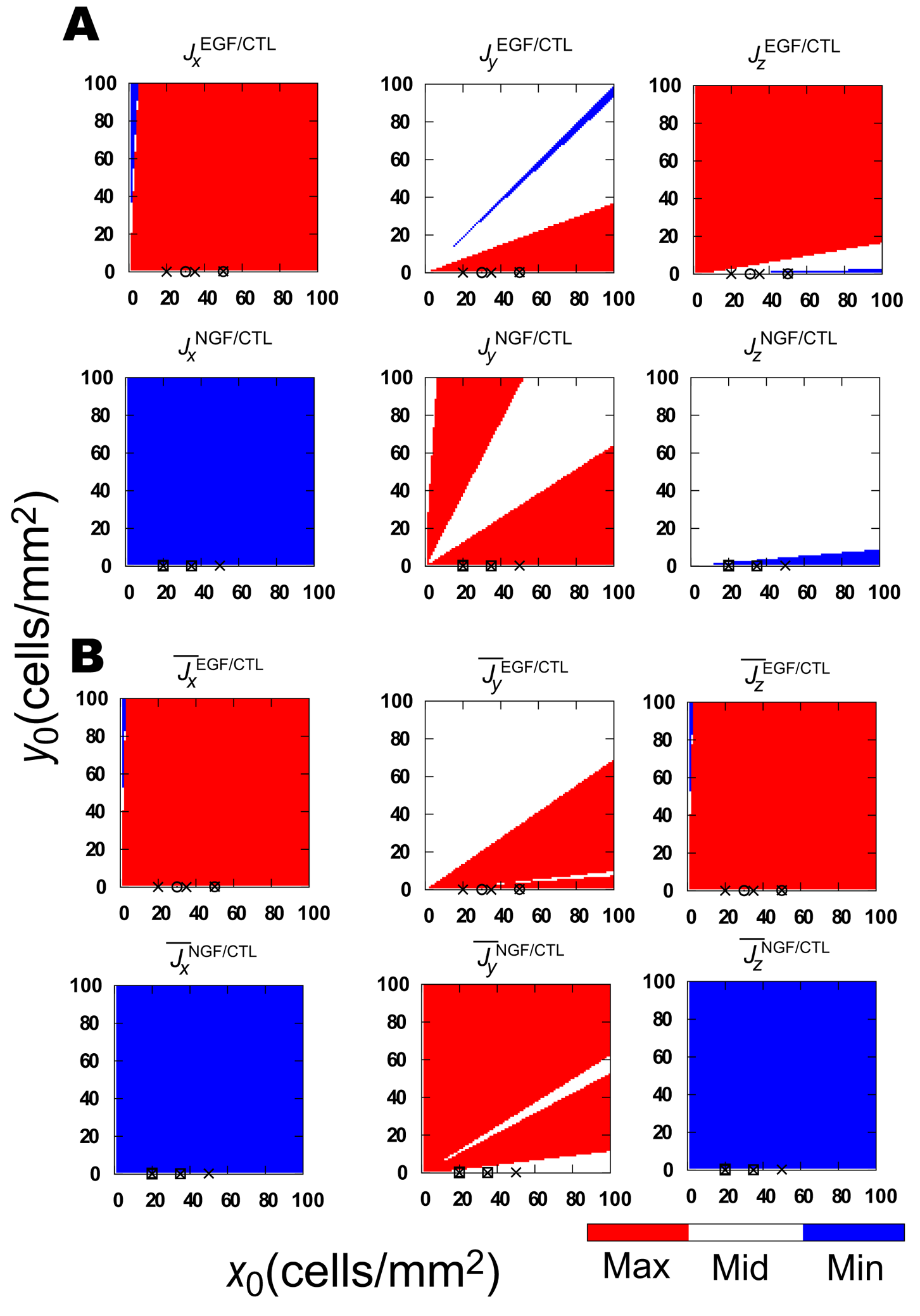

Supplement: Figure S4 — Dependency of response rates on initial conditions. The initial response speeds and () (A), or the time averages of response speeds and (B) for several initial conditions ( and ) and serum conditions (High serum: horse serum (HS) and fetal bovine serum (FBS); low serum: HS and FBS; serum free: bovine serum albumin.) were calculated. The initial condition do not affect those speeds. For each initial condition, we compared the speeds among three serum conditions. When the speed was maximal (minimal) in the middle entropy condition, we plotted a red (blue) point on a graph, respectively. When the speeds monotonically changed, the region of a graph is white. Initial conditions for our experimental results were also plotted on a graph (a cross mark for the control conditions, a circled mark for EGF-added conditions, and a box mark for NGF-added conditions). Estimated parameter values of experiment 1 in Table S1 was used for calculating these figures. (TIF) [file pcbi.1003320.s004.tif]

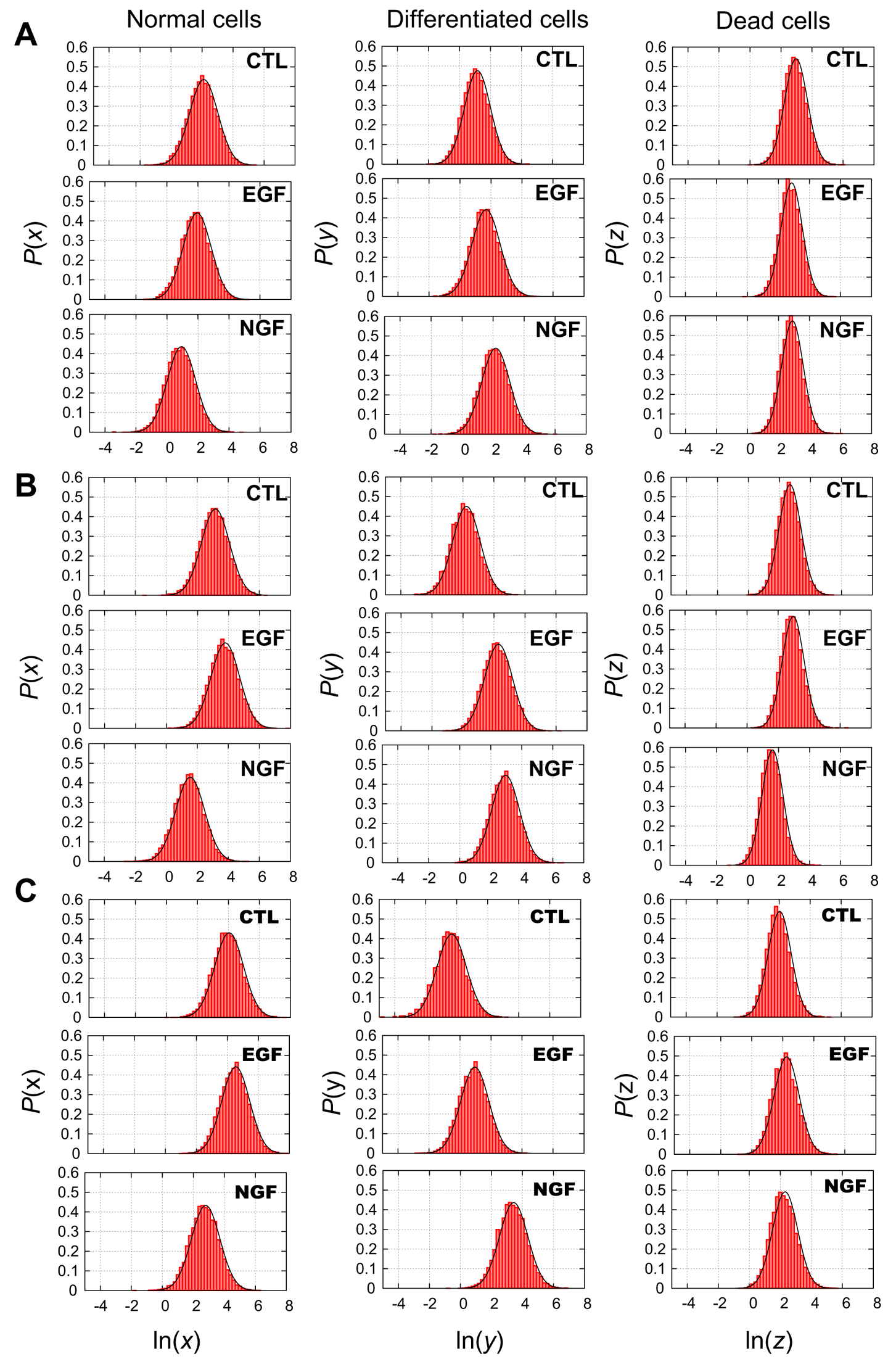

Supplement: Figure S5 — Log-normal distributions of the simulated cell density. Histograms of the cell densities (cells/mm2 ) at day are calculated using parameters in the serum free conditions (A), the low serum conditions (B), and the high serum conditions (C) in the presence or absence of growth factors (CTL, control). Simulations have done using the parameters in the high serum condition of model-1. Black lines denote normal distribution using means and variances from simulated data. (TIF) [file pcbi.1003320.s005.tif]

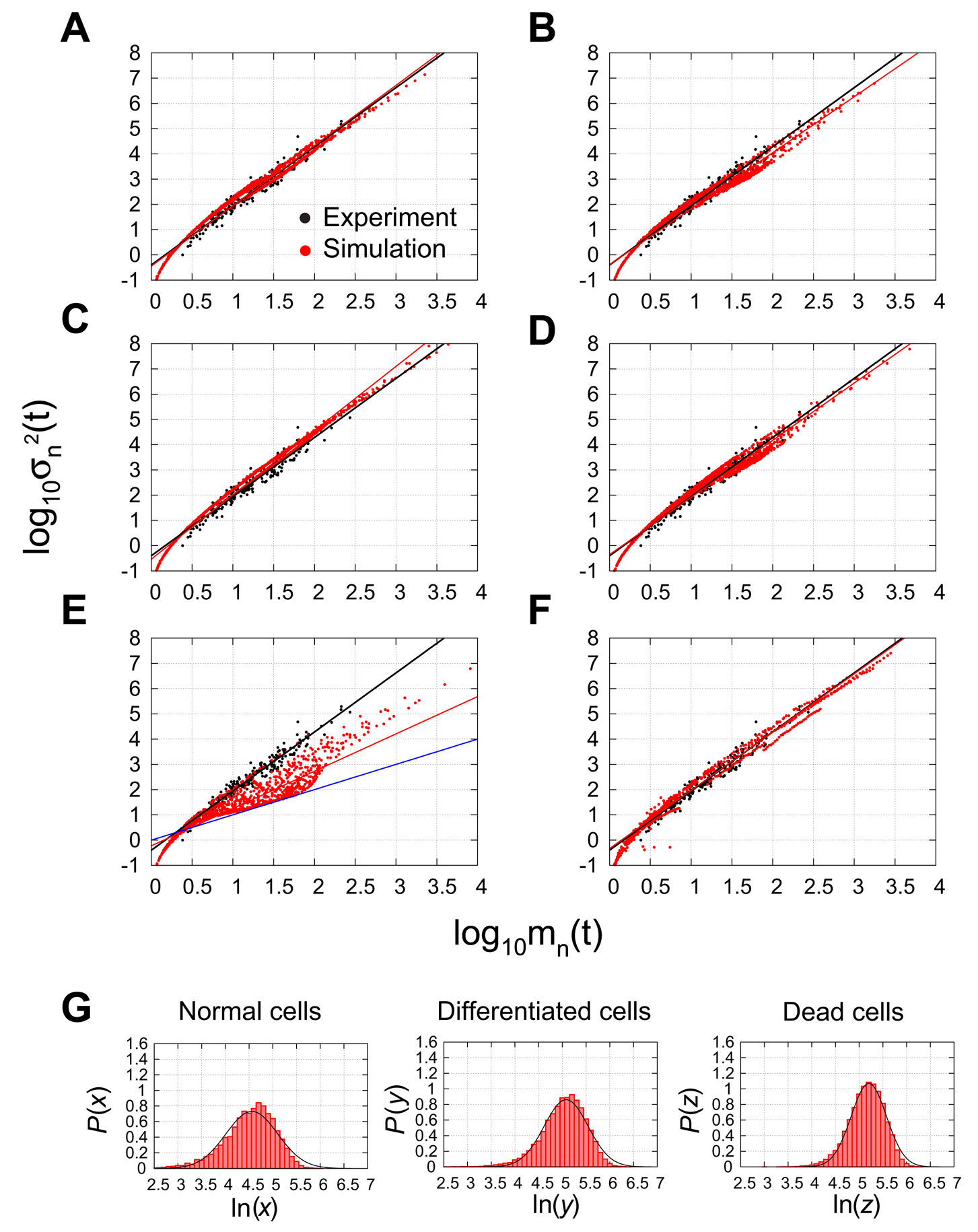

Supplement: Figure S6 — Power-law relations under various parameter values and initial conditions. To examine the generality of the result in Fig. 7, simulations were carried out changing the parameter values and initial distribution. (A–F) The relationship between mean and variance of cell density was calculated using parameter values independently selected from uniform random numbers with the range of . The mean initial numbers of cells were constant (A, C), or independently selected from (B, D–F). Initial distribution was set to obey lognormal (A, B), exponential (C, D), or Poisson (E, F) distribution. For the initial distributions, we used the equations (20) for a lognormal distribution, a function (where ) with (, , or ) for a exponential distribution, and a function (where is a natural number) with (, , or ) for a Poisson distribution. We simulated (methods were shown in models section), times with constant parameters and the initial number of cells to calculate means and variances at day (A–E, red circles) or day (F, red circles). We repeated times of this procedure with randomly sampled parameters or initial conditions, and estimated the slope for (red lines). The slopes were (A), (B), (C), (D), (E), and (F). The blue line in (E) denotes an equation with (Poisson distribution). In the figure (F), we used only and , and omitted values to evaluate a slope . (G) Distributions of cell density were calculated at day with arbitrarily defined parameter values and the mean initial conditions for a Poisson distribution. We simulated sample paths to make this distribution. As shown here, power-law relation did not depend on the specific conditions of parameter values and the initial number of cells when the initial distribution was lognormal and exponential. For Poisson distribution, the slope initially gradually increased to (E, F). In addition, the distribution of the number of cells became lognormal even when the initial number of cells obeyed a Poisson distribution (G). Therefore, the po [file pcbi.1003320.s006.tar]

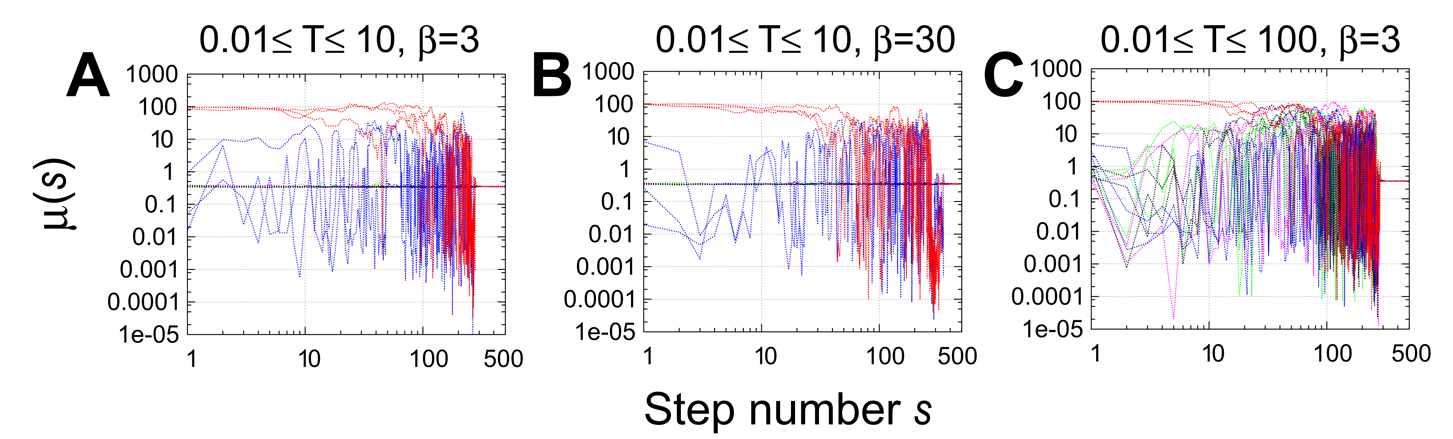

Supplement: Figure S7 — Dependency of parameter estimations on the initial conditions. To show the typical process of parameter estimations under different initial conditions, these figures plotted selected parameter values with the maximum likelihood in a constant temperature at each simulation step . We used experimental data from experiment 2 of high serum and control condition in Table S1. The gamma distributions with parameters , and (A, C) or (B) were used as the prior distribution. For the initial temperature, we defined (A, B) or (C). We selected the final temperature as for all figures. The initial parameter values in step and at time were or , where . In each figure, we showed three sample paths with the same initial conditions. In all cases, a wide range of values was searched in estimation and converged to similar values when . The parameter converged to (A), (B), and (C). (TIF) [file pcbi.1003320.s007.tif]
