# Supplementary material for: Optimality Conditions for Cell-Fate Heterogeneity That Maximize the Effects of Growth Factors in PC12 Cells
Source: PLoS Comput Biol. 2013 Nov 14;9(11):e1003320. doi: 10.1371/journal.pcbi.1003320 (PMC3828137; doi:10.1371/journal.pcbi.1003320)
Supplement: Table S1 — Estimated parameter values in four independent experiments. High serum: HS and FBS, low serum: HS and FBS, serum free: BSA. The initial conditions () for each serum and stimulus condition did not strictly affect estimation, and we arbitrarily assigned several values which could be estimated from experimental results. Values were estimated on the basis of the results shown in Figure 2 in the main text. Estimations were done for the independent four experimental results of the time courses of the number of cells. (PDF) [file pcbi.1003320.s008.pdf]

Experiment 1

Serum	Stimulus	μ	k_1	k_2	d_1	d_2	x_0	y_0	z_0
High	Control	0.316	0.0363	0.491	0.00811	2.07	20.0	0.1	2.0
	EGF	0.339	0.121	3.79	0.0263	0.254	30.0	0.2	1.0
	NGF	0.549	0.518	2.84E-04	0.00108	0.105	20.0	0.1	1.0
Low	Control	0.0766	0.0120	7.42E-04	0.0713	0.0721	35.0	0.05	5.0
	EGF	0.154	0.0725	0.00156	0.0361	0.169	50.0	0.2	7.0
	NGF	0.262	0.481	2.01E-04	0.00121	0.0341	20.0	0.5	3.0
Free	Control	1.10E-04	0.0424	4.95E-04	0.176	0.0360	50.0	0.3	5.0
	EGF	7.14E-05	0.120	0.105	0.140	0.0118	30.0	0.05	8.0
	NGF	6.74E-05	0.385	0.114	0.219	0.0394	35.0	0.2	5.0

Experiment 2

Serum	Stimulus	μ	k_1	k_2	d_1	d_2	x_0	y_0	z_0
High	CTL	0.352	0.0240	0.00243	0.0561	0.345	30.0	0.3	1.0
	EGF	0.386	0.0739	0.00226	0.00488	0.515	20.0	1.0	1.0
	NGF	0.751	0.592	4.93E-04	0.128	9.77E-04	15.0	0.4	2.0
Low	CTL	0.0860	0.0175	5.50E-04	0.100	0.0135	40.0	1.0	5.0
	EGF	0.186	0.142	2.27E-04	0.00356	0.227	40.0	0.5	7.0
	NGF	0.327	0.601	5.98E-04	0.111	7.30E-04	40.0	1.0	3.0
Free	CTL	6.79E-05	0.140	0.210	0.168	0.00127	40.0	0.5	5.0
	EGF	0.196	0.304	3.11E-04	0.00440	0.438	45.0	2.0	1.0
	NGF	0.183	0.600	0.0438	0.0307	0.0884	35.0	0.5	5.0

Experiment 3

Serum	Stimulus	μ	k_1	k_2	d_1	d_2	x_0	y_0	z_0
High	CTL	0.531	0.0251	0.00204	0.0370	5.39E-04	60.0	3.0	1.0
	EGF	0.557	0.0802	1.11	0.0329	0.00636	50.0	1.0	2.0
	NGF	0.674	0.682	0.0276	0.0167	0.0324	50.0	10.0	6.0
Low	CTL	0.181	0.0155	0.0608	0.0709	4.16E-04	55.0	0.0	3.0
	EGF	0.233	0.0874	2.07E-04	0.0164	0.416	50.0	1.0	0.0
	NGF	0.501	0.769	6.87E-06	0.0346	0.0363	35.0	10.0	0.0
Free	CTL	0.0569	0.0631	4.58E-04	0.147	1.40E-04	60.0	2.0	1.0
	EGF	0.177	0.221	4.95E-04	0.0605	0.507	65.0	1.0	1.0
	NGF	0.0301	0.368	8.91E-05	1.51E-05	0.178	80.0	5.0	0.1

Experiment 4

Serum	Stimulus	μ	k_1	k_2	d_1	d_2	x_0	y_0	z_0
High	CTL	0.497	0.0682	0.563	0.0363	0.00305	20.0	0.0	0.0
	EGF	0.726	0.0613	6.93E-04	0.0521	2.42E-04	15.0	0.0	0.5
	NGF	0.300	0.425	0.0229	0.0817	2.80E-05	30.0	0.0	1.0
Low	CTL	0.307	0.0579	0.00187	0.0846	0.859	40.0	0.0	0.0
	EGF	0.532	0.0859	2.96E-04	0.135	1.68E-04	25.0	0.5	1.0
	NGF	0.513	0.799	4.44E-05	0.0384	0.0334	30.0	0.0	1.0
Free	CTL	0.239	0.278	2.73E-04	0.0125	0.963	55.0	0.0	3.0
	EGF	0.178	0.306	9.69E-05	1.74E-05	0.590	50.0	2.0	0.0
	NGF	7.18E-05	1.36	0.468	0.00253	0.142	25.0	20.0	1.0
